# The Temporal and Spatial Invasion Genetics of the Western Corn Rootworm (Coleoptera: Chrysomelidae) in Southern Europe

**DOI:** 10.1371/journal.pone.0138796

**Published:** 2015-09-25

**Authors:** Darija Lemic, Katarina M. Mikac, Stephanie A. Ivkosic, Renata Bažok

**Affiliations:** 1 Department for Agricultural Zoology, University of Zagreb, Faculty of Agriculture, Zagreb, Croatia; 2 Centre for Sustainable Ecosystem Solutions, University of Wollongong, Wollongong, New South Wales, Australia; Queen's University Belfast, UNITED KINGDOM

## Abstract

This study describes the genetics of the western corn rootworm (WCR), *Diabrotica virgifera virgifera* LeConte in southern Europe during the introduction (1996–2001) and establishment/spread (2002–2011) phases of its invasion. The *Diabrotica* microsatellite core-set was used to perform traditional population genetics analyses. Our results indicated that during the introduction phase genetic diversity and population genetic structure were lower overall as compared to the establishment/spread phase. Unusually high genetic differentiation was found between the Italy and southern Europe comparisons, including high differentiation between Italian populations separated by a short distance during the establishment/spread phase. STRUCTURE analysis revealed two genetic clusters during the introduction phase and two genetic clusters during the establishment/spread phase. However, bottlenecked populations were only detected during the invasion phase. A small but significant isolation by distance effect was noted in both phases. Serbia was the geographic source of WCR to Croatia and Hungary in the introduction phase, while the United States of America was the possible source of WCR to Italy in 2001. These introductory populations were the subsequent source of individuals sampled during the establishment/spread phase. Repeated introductions and admixture events in southern Europe may have resulted in genetically diverse WCR populations that have attained 83% of all known alleles worldwide.

## Introduction

It is suspected that the western corn rootworm (WCR), *Diabrotica virgifera virgifera* LeConte (Coleoptera: Chrysomelidae) began its invasion of Europe ca. 1980, but the beetle was not officially recorded until 1992 from a maize field near Belgrade, Serbia [1]. Since 1998, at least five separate introductions of the WCR from North America into Europe are known to have occurred [2,3,4].

Similar to other invasive species [5], the invasion of Europe by the WCR has been characterized by three phases over the last two decades. 1. The accidental introduction of WCR into Europe which occurred ca. 1980–1992 [6,7]. 2. The establishment of the species in countries surrounding the geographic introduction location ca. 1995–2000 (i.e., countries neighbouring Serbia such as Croatia, Bosnia and Herzegovina, Hungary and Romania [7]). 3. The dispersal phase in which the WCR spread west from the locations where WCR first established to now 22 European countries spanning some tens thousands of hectares of arable land [7]. Whether the temporal invasion phase delineations broadly described above exist in those year ranges is a matter of further discussion. This is because it is difficult to decide precise cut-off dates for each of the invasion phases listed, as introductions from the dispersal of established populations are continuing (e.g., most recent detection of WCR was in Russia in 2011 [7]). From 1995 and up until 2001 newly invaded fields by WCR were routinely identified in southern Europe. In subsequent years (after 2002–2011) population density was stable in all maize growing areas, also reproduction (indicator of an established population [8]) was stable and density was increasing. Evidently, WCR has been established and spreading since ca. 1995 and as such the invasion phases of establishment and spread are likely to co-exist in southern Europe.

The WCR is a univoltine species that overwinters in the soil only to emerge in spring, and commence feeding upon the roots of maize plants damaging key plant physiological processes [9]. The resulting damage leads to lodging and yield losses that culminate in economic levels of damage to maize crops. Such losses have now been recorded in 22 European countries of which Croatia, Serbia and Hungary have suffered the most economic losses to date. Baufeld and Enzian [10] estimate damage and control costs of €147 per year in Europe while in its native region (USA) costs was estimated to be >US$ 1 billion per year [11]. The importance of understanding the invasion biology and genetics of a pest species is now fully recognised, and research carried out to that effect assists in the development and implementation of integrated management practises [12,13,14].

The *Diabrotica* genetics consortium was instrumental in recognizing the importance that molecular markers such as microsatellites could play in informing pest management strategies through accurate estimates of population genetic structure, gene flow and dispersal [15,16]. Hence, a core-set of microsatellite markers was developed for the WCR [17] from which it was possible to obtain solid population genetic data, including: estimates of population genetic structure; modes of dispersal and gene flow; geographic source of introductions [2,3,18]; and answers to more complex inter-continental (Europe vs. USA) invasion scenarios using approximate Bayesian computations [2,3,19]. While these population genetic studies have significantly improved our understanding of WCR invasions in Europe, apart from Bermond et al. [19] and Lemic et al. [18], there has been little attention paid to the population genetic status of the WCR in a particular European country and how genetic data can be applied for practical on-ground management purposes. This is important because if the WCR is to be forced (by way of integrated management) into fragmented/isolated populations (demonstrated through moderate (0.05–0.15) to large F_ST_ (>0.15) values) with restricted gene flow (e.g., red imported fire ant, *Solenopsis invicta* Buren in Australia: [20]), then the best way to achieve this is via mandated country specific quarantine, management and control practises. The work detailed herein was conducted to translate genetic information into practical on-ground management purposes in order to effectively control its global spread.

Using F-statistics, Lemic et al. [18] focused on estimating the historical and contemporary genetic structure of WCR in Croatia from 1996–2009, in a bid to establish baseline genetic data and information regarding WCR population genetics that could inform management practises. The aforementioned study yielded important allelic information including estimates of genetic structure and patterns of gene flow all while providing a much needed temporal comparison of WCR populations. The most useful results were those indicating that a single large WCR population existed in Croatia in both 1996 and 2009. However, the alleles and allele frequencies of the single large population changed over time as a result of admixture. The admixture event(s) effectively caused the significant difference in population genetic structure and difference in alleles found between the 1996 and 2009 populations. Such information is critical to the success of WCR management in Croatia [18] and elsewhere, because it informs management practises and management can focus on locations where estimates of genetic structure are higher and populations are beginning to fragment and isolate. It is in such locations that control must be intensified, further facilitating fragmentation in a bid to cause local extinctions or in the least local population suppression. In the absence of population genetic markers such as microsatellites, it would be impossible to detect the genetic effects that control is having on populations in the field.

While there are now numerous studies on the invasion genetics of WCR both in the USA [21] and Europe [2–4,19], none of these studies’ foci was applied. That is, the genetic data generated were not used to assist in the monitoring of existing and new populations or to inform or evaluate management practises by a country or by a region. We recognised this and in Lemic et al. [18] used an applied population genetics approach to investigate the WCR problem in Croatia and in doing so were able to provide genetic data with practical application in the monitoring and management of WCR, and provide recommendations for future management. The recommendations included elucidating source populations, a national spatial and temporal allelic inventory and open access digital repository of the genetic data. In our study we used samples from the USA for comparative purposes and to possibly detect the geographic source of southern European populations. Comparisons with USA samples had starting point in a survey by Kim and Sappington [21,22] where they showed no genetic differentiation among populations and high genetic similarity across the USA. Our present study builds upon our previous work with the aim to describe the invasion genetics (using traditional population genetics) of southern Europe over space and time with a focus on the stages of the invasion, broadly summarised from the literature as the introduction (1996–2001) and establishment/spread (2002–2011) phases. The specific aims of our current work are to: 1. infer and compare genetic diversity and demographic history among populations; and 2. identify the possible source of WCR during the introductory and establishment/spread phases of invasion in southern Europe. This work was conducted to further inform management practises and contribute to data for future genetic monitoring in Croatia and southern Europe.

## Materials and Methods

### Ethics statement

Western corn rootworm is an established pest of maize in Southern Europe. No special permission was needed for its collection in this study.

### Sample Collection

**Fig 1:**
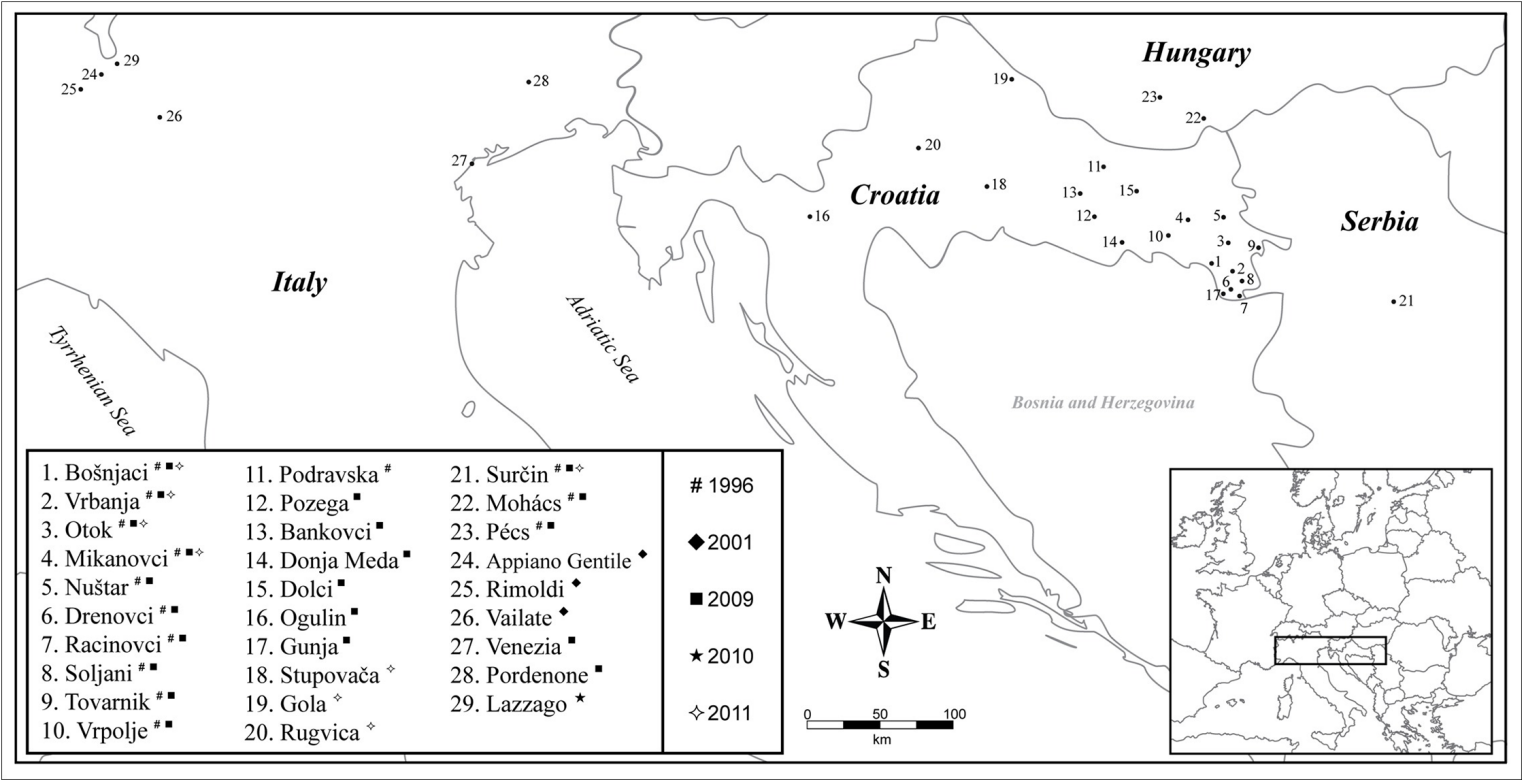
Locations of the WCR sampled in Croatia, Hungary, Serbia, Italy and USA during the introduction (1996–2001) and establishment/spread phases (2002–2011) of their invasion of southern Europe.

Adult WCR were collected from putative populations by hand throughout maize growing regions in Croatia, Hungary, Serbia and Italy during the introduction phase (1996–2001) and again during the establishment/spread phase (2002–2011). WCR sampled in Arizona, Iowa and Illinois were from soybean-maize rotated crops. The WCR sampled from Illinois were rotation-resistant variants. WCR sampled from the Nebraska were from continuous maize (Table 1, Fig 1). All USA WCR samples were collected by sweep netting. USA WCR were used in this study for comparative purposes. Once sampled, individuals were placed in 95% ethanol pending genetic analysis. For the Southern European data we conducted preliminary data analyses (F_ST_ estimates, see below) which yielded low to no genetic differentiation within country/year and therefore we treated country as a putative population (Table 1). The only exception was the Italy 2009 population which, based on high F_ST_ estimates, was categorised as its own population initially and in further analyses was further categorised by district e.g., Venezia district and Pordenone district.

**Table 1:** Country, sampling year, number (*n*) of locations and western corn rootworm (WCR) individuals sampled.

Country	Year	*n* locations	Locations	*n* WCR
Croatia	1996	11	Bošnjaci (Bos), Drenovci (Dre), Nuštar (Nus), Otok (Oto), Račinovci (Rac), Soljani (Sol), Mikanovci (Mik), Tovarnik (Tov), Vrbanja (Vrb), Vrpolje (Vrp), Podravska Moslavina (PM)	256
	2009	16	Bankovci (Ban), Bošnjaci, Donja Međa (DM), Dolci (Dol), Tovarnik, Drenovci, Nuštar (Nus), Otok, Račinovci, Soljani, Mikanovci, Vrbanja, Vrpolje, Ogulin (Ogu), Gunja (Gun), Požega (Poz)	495
	2011	7	Bošnjaci, Otok, Gola, Mikanovci, Rugvica, Vrbanja, Stupovača	111
Hungary	1996	2	Mohács (Moh), Pécs (Pec)	40
	2009	2	Mohács, Pécs	55
Serbia	1996	1	Surčin (Sur)	15
	2009	1	Surčin	30
	2011	1	Surčin	15
Italy	2001	3	Appiano Gentile (Apg), Rimoldi (Rim), Vailate (Vai)	60
	2009	1	Venezia (Ven)	30
	2009	1	Pordenone (Por)	30
	2010	1	Lazzago (Laz)	20
USA	2009	1	Arizona (AZ): Willcox (Will)	20
	2011	3	Iowa (IA): Nashua (Nas), Larrabee (Lar), Luther (Lut)	75
	2011	3	Illinois (IL): Urbana (Urb), Minonk, Dekalb	60
	2011	1	Nebraska (NE): Concord (Con)	15
**TOTAL**		**55**		**1327**

Location abbreviations in parentheses relate to S2 Table.

### Microsatellites

Total genomic DNA was isolated from individual *D*. *v*. *virgifera* using the Qiagen DNEasy extraction kit (Crawley, West Sussex) following the manufacturer’s guidelines. Multiplex polymerase chain reaction (MP-PCR) assays were conducted using the Qiagen MP-PCR kit (Crawley, West Sussex), again following the manufacturer’s guidelines. Kits were used to ensure that standard and uniform methods of DNA extraction and MP-PCR were conducted to facilitate data sharing as per the aims of the *Diabrotica* Genetics Consortium [14]. The amplification of microsatellite loci was conducted in a set of two MP-PCR assays where: MP1 amplified loci DVV-D2, DVV-T2 and Dba05; and MP2 amplified loci DVV-D4, DVV-D8 and Dba07 [15,23]. The PCR assay and cycling conditions used are outlined by Miller et al. (2007). Individual *D*. *v*. *virgifera* were genotyped using an Applied Biosystems (ABI) 3100 genetic analyzer (Foster City, CA). Raw genotypes were scored using GeneMapper v3.7 (ABI systems) (Foster City, CA). To standardize allele calling, reference samples from Arizona (USA) were genotyped and then alleles were scored according to data presented in Kim et al. [17]. DNA from all samples are housed in the Department for Agricultural Zoology, University of Zagreb and available from the authors upon request.

### Data Analysis

#### Genetic Diversity

Estimates of genetic diversity (i.e., mean number of alleles per locus (*A*), expected (H_E_) and observed (H_O_) heterozygosity, F_IS_ (inbreeding co-efficient)) per locus and population per phase of invasion, including tests of fit to Hardy-Weinberg equilibrium (HWE) and genotypic linkage equilibrium were estimated using GENEPOP on the Web v3.4 [24,25]. We compared *A* values between population samples by estimating allelic richness (*AR*) based on the smallest sample size using the rarefaction method in HP-RARE [26].

#### Genetic Differentiation

Population differentiation (i.e., F_ST_ / θ) was calculated among population pairs using FSTAT v2.9.3 [27]. GENEPOP on the Web v3.4 was then used to test for significant differentiation (Fisher exact tests) among populations for all loci and population pairs.

#### Genetic Clustering

The genetic clustering of individuals based on Bayesian methods was undertaken using the program STRUCTURE v2.3.3 [28]. Genetic clusters (*K*) were either set between one and seventeen (one more than the total number of populations for the complete data set) and a series of 10 replicate runs for each prior value of K were analysed. The parameter set for each run consisted of a burn-in of 10, 000 iterations followed by 1, 000, 000 MCMC iterations. The first inference performed, combined the admixture model of ancestry with the correlated allele frequency model of Falush et al. [29] and the sampling information. The second inference performed combined the admixture model of ancestry with the correlated allele frequency model without the sampling information [30]. For both inferences, the default parameters in STRUCTURE were maintained. To estimate K the Evanno et al. [31] method implemented in STRUCTURE HARVESTER v0.6.93 [32] was used, where the highest ΔK value was indicative of the number of genetic clusters. In addition, when estimating K, the proportion of the sample assigned to a population was checked for asymmetry (indicates population genetic structure) or symmetry (1/K = indicates no population genetic structure) [28]. The graphical program DISTRUCT v1.1 [33] was used to display the STRUCTURE output.

#### Population Bottleneck

The presence of population bottlenecks was assessed using BOTTLENECK v1.2. [34] via two models (i.e., stepwise-mutation model (SMM) and the two phase mutation model (TPM)). The TPM incorporates both the stepwise and multiple-step model. The TPM was run for 100,000 simulations with 90% SMM in TPM and 10% variance. The Wilcoxon signed-rank test was applied to determine significant deviations of excess heterozygosity across all loci relative to the expected drift-mutation equilibrium. Analysis of allele frequency distribution, to detect a mode-shift, was used as a further indicator of a population bottleneck [35]. These calculations were made for spatial comparisons among populations sampled during the introduction phase and the same populations in the establishment/spread phase in Croatia, Hungary, Serbia and Italy (Table 1). For final bottleneck analyses, populations were pooled by country and time period owing to the fact that within country pairwise F_ST_ values were very small and suggestive of a single large population per country.

#### Geographic Source

Identification of the most likely geographic source of populations was performed by calculating the mean individual assignment likelihood (L) for each individual (i) to the possible source population(s), referred to as Li→s [36,37], using GENECLASS v2.0 [38]. Li→s values were calculated using the Rannala and Mountain [36]) criterion, Paetkau et al. [39] simulation algorithm and 1,000,000 simulated individuals. The evaluation of the most likely geographic source population was determined in each sample based on the combined lowest F_ST_ and the highest Li→s value [39].

#### Isolation by Distance

Testing for isolation by distance was undertaken in FSTAT v2.9.3 [27] using a Mantel test to determine if there was a significant correlation between matrices of genetic differentiation (i.e., Slatkin’s [40] linearized F_ST_: F_ST_ [1/ F_ST_-1]) and the natural logarithm (ln) of geographic distance in kilometres (km). These calculations were made for spatial comparisons among populations sampled during the introduction (Croatia, Hungary, Serbia and Italy) and establishment/spread phases in the same region. An additional isolation by distance analysis was calculated without the Italian populations (from 2001, 2009 and 2010) in both phases to determine whether this pattern would hold if distant European populations were excluded from the analyses. Also, after identification of geographic source population of southern European populations, isolation by distance analysis was calculated without the identified source population to evaluate the role of source populations in the geographic distance analyses.

## Results

### Genetic Diversity

Of all known alleles worldwide (77 alleles) [3,17,18,21,22] 45% of them (35 alleles) were found in the introduction phase of WCR invasion in Europe, and 84% (65 alleles) were found during the establishment/spread phase. USA populations had 11 alleles not previously found in Europe. Nine of these alleles were found only in Croatia and the USA. Also, 13 alleles found were unique to Europe only and only two of these unique alleles were found during the introduction phase, and the rest were found during the establishment/spread phase. Microsatellite allele frequencies for each locus and population are detailed in S1 Table. Allelic richness per locus and mean allelic richness are presented in Table 2.

**Table 2:** Allelic information for the western corn rootworm sampled during the introduction (1996–2001) and establishment/spread phases (2002–2011). Values in bold indicate establishment/spread phases. USA samples represent ‘native populations’ and were used in this study for comparative purposes.

Population	Microsatellite Loci						Mean
	DVV-D2	DVV-T2	Dba05	DVV-D4	DVV-D8	Dba07	
**Croatia 1996**	n = 227	n = 251	n = 236	n = 245	n = 239	n = 238	239
*A*	8	3	2	5	9	5	5.33
*AR*	4.27	2.05	2.00	3.15	4.23	3.21	3.15
F_IS_	0.11***	-0.37***	-0.33***	-0.11***	0.009*	-0.07***	-0.13
H_O_	0.63	0.57	0.66	0.66	0.67	0.66	0.64
H_E_	0.71	0.41	0.49	0.60	0.68	0.61	58
**Hungary 1996**	n = 34	n = 40	n = 40	n = 40	n = 40	n = 40	39
*A*	5	2	2	3	4	3	3.2
*AR*	4.73	2.00	2.00	2.99	3.60	3.59	2.99
F_IS_	0.25	-0.26	-0.09	0.20	0.07	0.01	0.03
H_O_	0.50	0.60	0.47	0.50	0.45	0.65	0.53
H_E_	0.62	0.45	0.42	0.62	0.46	0.65	0.54
**Serbia 1996**	n = 15	n = 15	n = 15	n = 14	n = 13	n = 13	14
*A*	4	2	2	3	7	3	3.5
*AR*	3.99	2.00	2.00	3.00	7.00	3.00	3.5
F_IS_	0.29	-0.87*	-0.40	0.00	-0.04	-0.23	-0.20
H_O_	0.47	0.93	0.60	0.50	0.77	0.84	0.69
H_E_	0.60	0.47	0.40	0.50	0.69	0.69	0.56
**Italy 2001**	n = 47	n = 58	n = 56	n = 59	n = 56	n = 57	56
*A*	4	2	2	5	11	5	4.8
*AR*	2.74	2.00	1.99	2.66	6.77	3.04	3.2
F_IS_	0.65***	-0.40**	-0.20	0.06	0.05**	-0.10***	0.01
H_O_	0.06	0.57	0.43	0.47	0.75	0.60	0.48
H_E_	0.17	0.40	0.36	0.49	0.79	0.54	0.46
**Croatia 2009**	**n = 474**	**n = 472**	**n = 483**	**n = 474**	**n = 480**	**n = 482**	**478**
*A*	**9**	**6**	**2**	**8**	**13**	**6**	**7.3**
*AR*	**5.57**	**3.18**	**2.18**	**3.50**	**5,48**	**3,89**	**3.97**
F_IS_	**-0.14** ***	**-0.003**	**-0.18** ***	**0.04** **	**-0.05**	**-0.06**	**-0.07**
H_O_	**0.73**	**0.31**	**0.59**	**0.59**	**0.70**	**0.66**	**0.60**
H_E_	**0.64**	**0.31**	**0.50**	**0.61**	**0.66**	**0.62**	**0.56**
**Croatia 2011**	**n = 111**	**n = 111**	**n = 111**	**n = 111**	**n = 111**	**n = 111**	**111**
*A*	**10**	**7**	**6**	**5**	**10**	**4**	**7**
*AR*	**5.61**	**3.30**	**2.59**	**3.78**	**5.99**	**3.91**	**4.19**
F_IS_	**0.06** *	**-0.14**	**-0.12** ***	**-0.07**	**-0.08** *	**0.10**	**-0.04**
H_O_	**0.71**	**0.44**	**0.59**	**0.68**	**0.77**	**0.56**	**0.63**
H_E_	**0.76**	**0.38**	**0.51**	**0.63**	**0.70**	**0.63**	**0.60**
**Hungary 2009**	**n = 54**	**n = 55**	**n = 55**	**n = 53**	**n = 53**	**n = 54**	**54**
*A*	**5**	**3**	**2**	**4**	**7**	**6**	**4.5**
*AR*	**4.99**	**2.49**	**2.00**	**3.51**	**5.73**	**4.50**	**3.87**
F_IS_	**0.11** *	**-0.32** *	**-0.17**	**-0.03**	**0.13** *	**0.005**	**-0.04**
H_O_	**0.65**	**0.51**	**0.58**	**0.64**	**0.53**	**0.54**	**0.58**
H_E_	**0.72**	**0.38**	**0.49**	**0.60**	**0.60**	**0.54**	**0.56**
**Serbia 2009**	**n = 28**	**n = 30**	**n = 30**	**n = 30**	**n = 30**	**n = 30**	**30**
*A*	**7**	**3**	**2**	**3**	**6**	**4**	**4.2**
*AR*	**7.56**	**4.04**	**2.00**	**4.80**	**6.04**	**3.84**	**4.71**
F_IS_	**0.60** ***	**-0.87** ***	**-0.56** *	**0.10**	**0.21**	**-0.09**	**-0.10**
H_O_	**0.32**	**0.96**	**0.77**	**0.53**	**0.50**	**0.70**	**0.52**
H_E_	**0.79**	**0.50**	**0.47**	**0.57**	**0.60**	**0.63**	**0.59**
**Serbia 2011**	**n = 15**	**n = 15**	**n = 15**	**n = 15**	**n = 15**	**n = 15**	**15**
*A*	**6**	**4**	**2**	**6**	**5**	**3**	**4.3**
*AR*	**7.79**	**4.20**	**2.18**	**4.92**	**6.59**	**3.98**	**4.94**
F_IS_	**0.09**	**0.40** *	**0.20**	**-0.23**	**-0.03**	**0.20**	**0.10**
H_O_	**0.73**	**0.27**	**0.40**	**0.80**	**0.73**	**0.53**	**0.58**
H_E_	**0.80**	**0.40**	**0.47**	**0.60**	**0.67**	**0.60**	**0.59**
**Italy 2009**	**n = 53**	**n = 54**	**n = 54**	**n = 55**	**n = 55**	**n = 55**	**54**
*A*	**6**	**6**	**3**	**5**	**7**	**6**	**5.5**
*AR*	**5.71**	**5.54**	**2.85**	**4.58**	**6.44**	**5.20**	**5.05**
F_IS_	**0.28** ***	**-0.06** **	**-0.55** ***	**0.06** ***	**0.21** ***	**0.10** ***	**0.01**
H_O_	**0.47**	**0.46**	**0.78**	**0.45**	**0.60**	**0.64**	**0.57**
H_E_	**0.64**	**0.43**	**0.50**	**0.47**	**0.75**	**0.71**	**0.58**
**Italy 2010**	**n = 17**	**n = 19**	**n = 19**	**n = 20**	**n = 20**	**n = 20**	**19**
*A*	**6**	**3**	**2**	**3**	**8**	**3**	**4.2**
*AR*	**7.00**	**2.93**	**2.00**	**3.00**	**7.79**	**3.00**	**4.29**
F_IS_	**0.30** *	**-0.72** *	**-0.28**	**-0.28**	**0.09** *	**0.02**	**-0.16**
H_O_	**0.35**	**0.89**	**0.47**	**0.60**	**0.75**	**0.55**	**0.60**
H_E_	**0.47**	**0.52**	**0.37**	**0.45**	**0.80**	**0.55**	**0.53**
**USA 2009**	n = 20	n = 20	n = 20	n = 15	n = 20	n = 20	19
*A*	8	6	2	6	14	5	6.8
*AR*	7.17	5.25	2.00	6.00	12.54	4.88	6.31
F_IS_	0.12	-0.15	-0.04	0.33	-0.10	-0.10	0.01
H_O_	0.65	0.60	0.40	0.53	1	0.75	0.66
H_E_	0.70	0.50	0.35	0.73	0.90	0.65	0.64
**USA 2011**	n = 149	n = 149	n = 148	n = 150	n = 149	n = 150	149
*A*	11	4	4	10	21	7	9.5
*AR*	7.28	3.08	2.89	6.36	11.99	4.07	5.95
F_IS_	0.03	-0.21**	-0.006	0.04*	-0.03	-0.04*	-0.03
H_O_	0.72	0.68	0.51	0.67	0.92	0.68	0.70
H_E_	0.74	0.56	0.50	0.70	0.89	0.65	0.67

*A*: Number of alleles. *AR*: Allelic richness. F_IS_: Weir and Cockerham’s [43] inbreeding coefficient. H_O_: Observed heterozygosity. H_E_: Expected heterozygosity. Significant deviation from Hardy-Weinberg Equilibrium

* *P* < 0.05

** *P* < 0.01

*** *P* < 0.001

#### Introduction Phase

The mean number of alleles per locus *A* ranged from two (Dba05, DVV-T2) to 11 (DVV-D8) with an overall mean of 4.2 alleles per locus (Table 2). The largest mean number of alleles was found for the Croatia 1996 population(s) (5.33) compared to the other European populations surveyed (3.2–4.8) (Table 2). The standardization of *A* as a function of smallest sample size (*AR* in Table 2) made it possible to compare samples. In the introduction phase *AR* varied widely among samples; from 1.99 (Italy) to 7.00 (Serbia) (Table 2), with Serbia having the highest mean allelic richness of any other European location. Heterozygosity estimates (H_O_ and H_E_) over all loci and populations were very similar for all European populations. The average H_O_ per population ranged from 0.48 to 0.69, while the average H_E_ ranged from 0.46 to 0.58. F_IS_ estimates per locus and per population ranged from -0.87 (Serbia) to 0.65 (Italy) (Table 2).

The majority of populations examined did not significantly deviate from HWE. Deviations from HWE were not found at any loci for putative populations from Hungary. The significant deviations from HWE found in Croatia (DVV-T2, Dba05, DVV-D4, Dba07), Italy (DVV-T2, Dba07) and Serbia (DVV-T2) were associated with negative F_IS_ values, indicative of an excess of heterozygotes (Table 2). Conversely, the significant deviations from HWE present in Croatia (DVV-D2) and Italy (DVV-D2, DVV-D8) were associated with positive F_IS_ values indicative of an excess of homozygotes.

#### Establishment/Spread Phase

The mean number of alleles per locus ranged from two (Dba05) to 13 (DVV-D8) with an overall mean of 5.3 alleles per locus (Table 2). Overall the largest mean number of alleles was found in the USA (6.8–9.5). In southern Europe the largest mean number of alleles was found in Croatia (7.3). In the establishment/spread phase *AR* was highly variable and ranged from 2.00 (Hungary, Serbia, Italy) to 7.79 (Serbia, Italy) (Table 2). Heterozygosity estimates (H_O_ and H_E_) over all loci and populations were similar in all southern European populations, and comparable to the introduction phase. The average H_O_ per population ranged from 0.57 to 0.63, while average H_E_ ranged from 0.53 to 0.68. F_IS_ estimates per locus and per population ranged from -0.162 to 0.103 (Table 2).

Significant deviations from HWE were found in: Croatia for DVV-D2, Dba05 and DVV-D8; Hungary for DVV-T2; Serbia for DVV-T2 and Dba05; and Italy for DVV-T2 and Dba05. These deviations were also all associated with negative F_IS_ values. Conversely, significant deviations from HWE that were present in: Croatia for DVV-D4 and DVV-D2; Hungary for DVV-D2; Serbia for DVV-D2 and DVV-T2; and Italy for DVV-D2, DVV-D4, DVV-D8 and Dba07, were all associated with positive F_IS_ values.

Heterozygosity estimates over all loci and populations were very similar in southern Europe and the USA. The average H_O_ per population ranged from 0.52 to 0.60, while average H_E_ ranged from 0.53 to 0.59. F_IS_ estimates per locus and per population ranged from -0.87 (Serbia) to 0.60 (Serbia). Significant deviations from HWE were noted at only three loci from the 2011 USA population, and were associated with negative F_IS_ values (Table 2).

### Genetic Differentiation

We conducted preliminary data analyses (F_ST_ estimates, see below) which if they yielded low to no genetic differentiation within country/year and we subsequently treated country as a single putative population (Table 1). The only exception was the Italy 2009 population which because of high F_ST_ estimates was categorised as its own population initially and in further analyses was separated into districts (e.g., Venezia district and Pordenone district).

#### Introduction Phase

Pairwise comparisons of F_ST_ indicated a large range in population differentiation (F_ST_ = 0.014–0.164, *P* < 0.05) (S2 Table, Table 3). Non-significant low levels of differentiation were found within the first introduction of WCR into Croatia, Hungary and Serbia. When Italy was compared to these countries significant levels of differentiation (F_ST_ = 0.11–0.16, *P* < 0.05) were found (S2 Table, Table 3).

**Table 3:** Pairwise estimates of F_ST_ (θ: [41]) and mean individual assignment of likelihood (*L*_i→S_) of each potential source population (indicated in parentheses) [38] sampled during the southern European introduction and establishment/spread phases. Samples from the USA were included as the USA is the known source of WCR in Europe [2].

USA					Southern Europe				Southern Europe								
					Introduction Phase				Establishment/Spread Phase								
	Arizona2009	Iowa 2011	Illinois2011	Nebraska2011	Serbia 1996	Croatia1996	Hungary 1996	Italy 2001	Serbia2009	Serbia 2011	Croatia2009	Croatia 2011	Hungary 2009	Italy 2009*	Italy 2009**	Italy 2010	Potential source population
**Serbia, 1996**	0.13 (10.60)	0.06 (6.00)	0.06 (6.14)	0.06 (7.36)	-												**-**
**Croatia, 1996**	0.15 (11.49)	0.07 (6.28)	0.07 (6.32)	0.06 (7.16)	**0.01 (4.29)**	-											**Serbia 1996**
**Hungary, 1996**	0.17 (12.57)	0.09 (6.67)	0.10 (6.97)	0.11 (8.02)	**0.01 (4.35)**	0.03 (4.16)	-										**Serbia 1996**
**Italy, 2001**	0.18 (8.82)	**0.11 (5.64)**	**0.12 (5.98)**	0.14 (7.46)	**0.11 (5.92)**	0.13 (8.88)	0.16 (8.34)	-									**USA (Iowa-Illinois)/Serbia 1996**
**Serbia, 2009**	0.16 (13.05)	0.07 (6.93)	0.09 (7.38)	0.07 (8.20)	0.03 (5.74)	0.03 (5.92)	**0.01 (5.09)**	0.18 (9.24)	-								**Hungary 1996**
**Serbia, 2011**	0.16 (14.67)	0.09 (7.91)	0.09 (8.06)	0.07 (8.80)	0.08 (6.36)	0.03 (6.35)	0.04 (6.13)	0.22 (11.64)	0.03 (6.24)	-							**Croatia 2011** ***
**Croatia, 2009**	0.18 (12.94)	0.11 (6.86)	0.10 (6.90)	0.09 (8.19)	0.06 (5.04)	0.03 (4.63)	0.03 (4.53)	0.15 (8.84)	0.04 (4.65)	0.02 (6.27)	-						**Hungary 2009** ***
**Croatia, 2011**	0.16 (14.06)	0.09 (7.34)	0.09 (7.48)	0.07 (8.37)	0.06 (5.65)	0.02 (5.31)	0.04 (5.36)	0.17 (10.98)	0.04 (5.62)	**0 (5.64)**	0.02 (5.33)	-					**Serbia 2011** ***
**Hungary, 2009**	0.17 (12.68)	0.09 (6.58)	0.09 (6.79)	0.08 (7.84)	0.05 (4.97)	0.02 (4.56)	0.02 (4.45)	0.17 (8.89)	0.02 (4.71)	0.01 (5.94)	**0.01 (4.37)**	0.01 (5.12)	-				**Croatia 2009** ***
**Italy, 2009** *	0.16 (14.78)	0.09 (11.71)	0.09 (12.48)	0.10 (12.54)	0.11 (6.41)	0.14 (11.01)	0.17 (9.82)	**0.04 (4.53)**	0.16 (8.36)	0.20 (13.09)	0.15 (9.75)	0.17 (12.45)	0.17 (8.86)	-			**Italy 2001**
**Italy, 2009** **	0.20 (13.85)	0.15 (8.02)	0.14 (8.30)	0.16 (9.70)	0.09 (6.31)	0.08 (6.43)	0.07 (5.32)	0.24 (9.16)	**0.06 (4.94)**	0.09 (8.70)	0.08 (5.73)	0.08 (8.63)	0.08 (5.40)	0.22 (9.89)	-		**Serbia 2009** ***
**Italy, 2010**	0.15 (10.14)	0.07 (6.08)	0.09 (6.62)	0.09 (8.32)	0.04 (5.93)	0.09 (9.07)	**0.01 (8.45)**	0.02 (4.90)	0.01 (7.78)	0.16 (10.08)	0.12 (8.73)	0.13 (9.55)	0.12 (8.33)	0.02 (5.43)	0.17 (8.23)	-	**Italy 2001**

**Criteria for determining population source is lowest F**_**ST**_ **and highest *L***_**i→S**_ **(bold values).**

*Venezia district

**Pordenone district

***admixed population of southern Europe (originating from the first populations introduced in Europe.

Significant levels of differentiation were found at the intercontinental spatial scale between all southern European and USA populations (F_ST_ = 0.06–0.20, *P* < 0.05). Non-significant and low levels of genetic differentiation were found between pairwise USA population comparisons despite geographical distances separating some population comparisons of 100–2500 km (Table 3).

#### Establishment/Spread Phase

Pairwise comparisons of F_ST_ [41] indicated low to high levels of differentiation depending on the spatial scale of the comparison (F_ST_ = -0.007–0.22, *P* < 0.05) (S2 Table, Table 3). Significant but low levels of differentiation were found at a regional spatial scale (15 – 350 km within southern European populations [F_ST_ = -0.007–0.03, *P* < 0.05]). Alternatively, significant yet moderate levels of differentiation (F_ST_ = 0.167–0.22, *P* < 0.05) were found between southern European populations and Italian populations. Also, large and highly significant levels of differentiation (F_ST_ = 0.22, *P* < 0.01) were found among Italian populations sampled in 2009 (S2 Table, Table 3) despite a relatively short distance of 50 km separating them (i.e., Friuli Venezia Giulia [Venezia district] and Cordenons [Pordenone district]).

### Genetic Clustering

#### Introduction Phase

**Fig 2:**
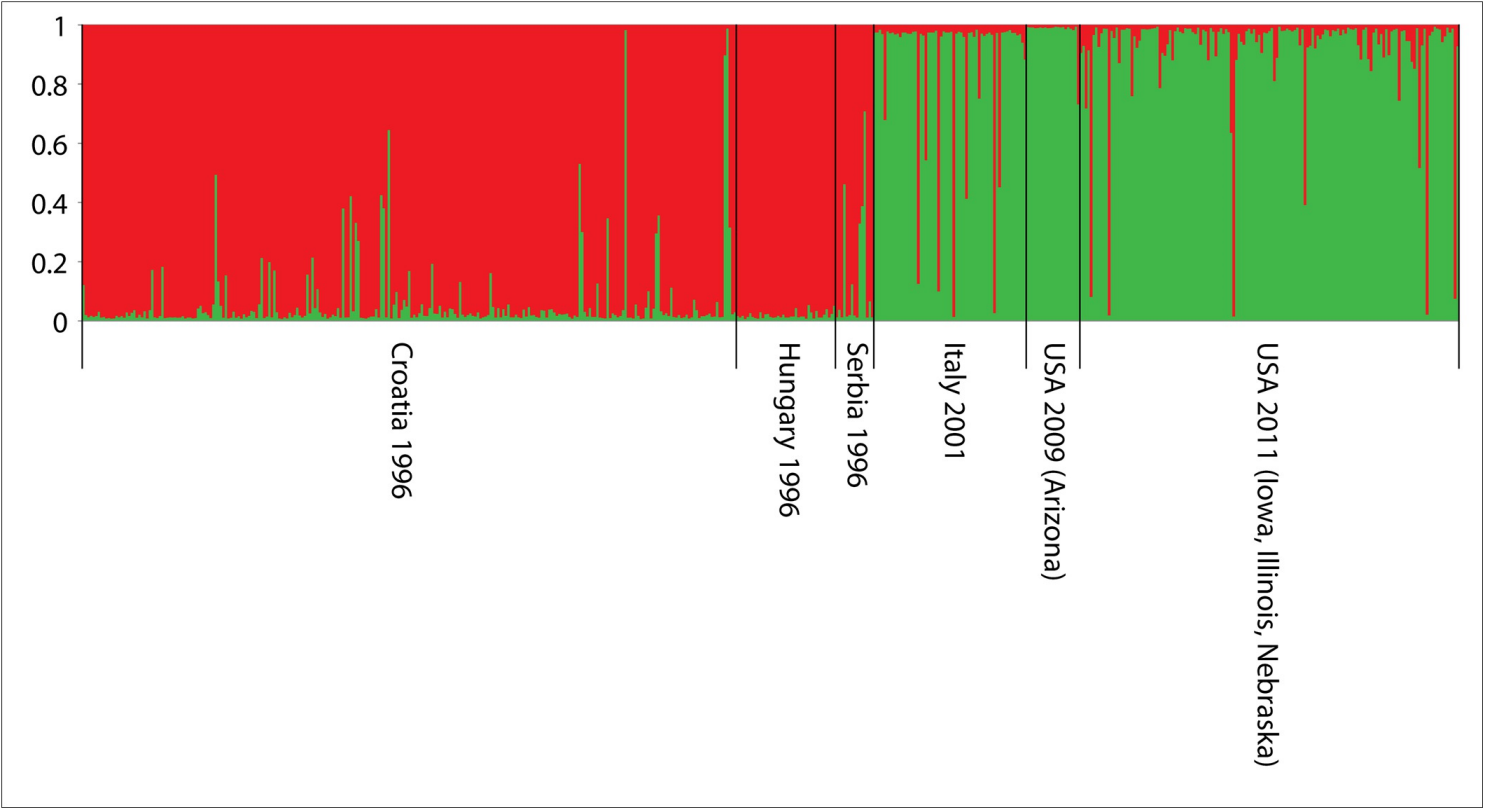
Genetic clustering of the first introduced populations (1996–2001) sampled in (1) Croatia, (2) Hungary, (3) Serbia and (4) Italy and putative native populations from the USA (5, 6) as a comparison.

Bayesian genetic clustering methods using STRUCTURE detected two distinct genetic clusters (*K* = 2, Fig 2, S2 Fig). The first cluster included all the southern European populations, while the second cluster included all the US populations, which were included for comparative purposes. In support of this result, the likelihood analysis based on Δ*K*, which was used to detect the actual number of clusters, confirmed that two genetic clusters (Δ*K* = 2) existed among the first population(s) introduced into southern Europe that arrived from the USA (S1A Fig).

#### Establishment/Spread Phase

**Fig 3:**
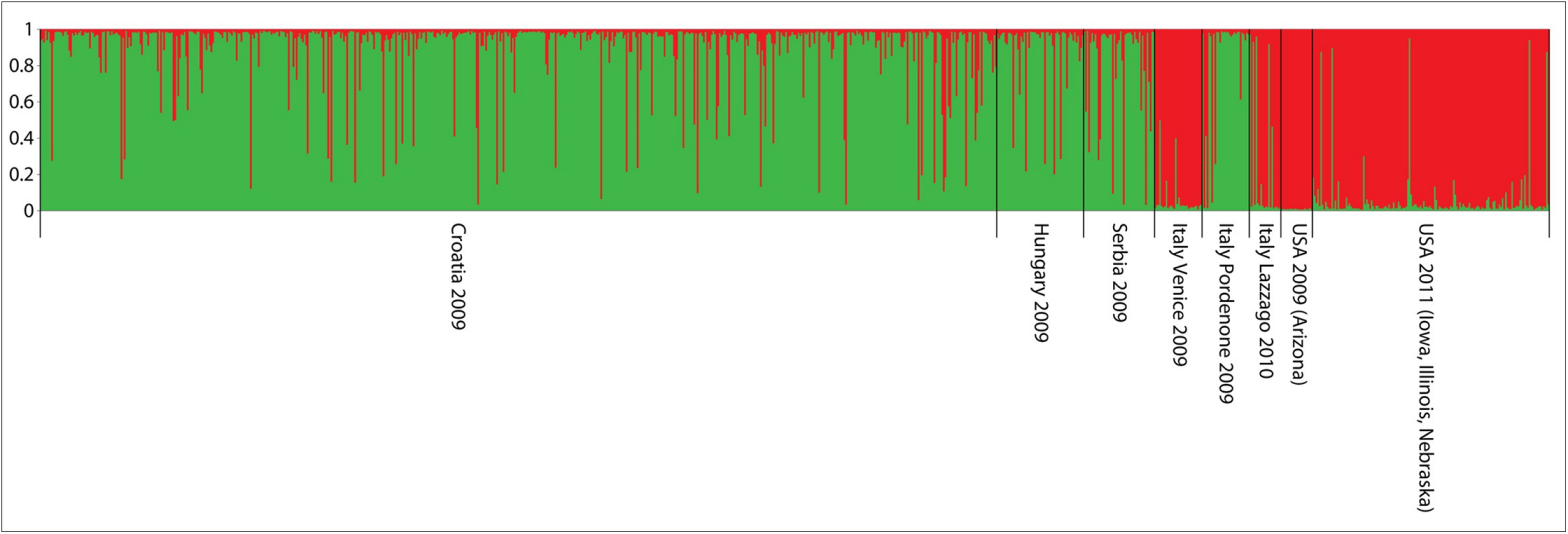
Genetic clustering of the established/spreading populations sampled in: (1) Croatia (2009); (2) Hungary (2009); (3) Serbia (2009); three locations from Italy, (4) Friuli Venezia Giulia (2009), (5) Cordenons (Pordenone region) (2009), (6) Lazzago (2010); and the USA (Arizona, Iowa, Illinois, Nebraska).

For populations collected during the establishment phase in Europe (2009–2011) and the US populations (sampled for comparative purposes) Bayesian genetic clustering detected two distinct genetic clusters (*K* = 2, Fig 3). The first cluster included populations from the first invaded area in Europe (Serbia, Croatia and Hungary) and one Italian population (Pordenone region), while the second cluster comprised all the US populations and other Italian population (Friuli Venezia Giulia [Venezia district]) (Fig 2). In support of this the likelihood analysis based on Δ*K* confirmed that two genetic clusters (Δ*K* = 2) existed among the inter-continental population comparison made (S1B Fig).

### Population Bottleneck

#### Introduction Phase

During the introduction phase Hungary and Serbia had a significant heterozygosity excess as indicated by the Wilcoxon sign-rank test (*P* < 0.05). In addition, Hungary and Serbia both experienced bottleneck events during the introduction phase, as indicated by a significant shifted mode non-normal distribution of alleles (*P* < 0.05). Croatia and Italy had a normal L shaped distribution of alleles, hence no indication of a bottleneck event.

#### Establishment/Spread Phase

In contrast, during the establishment phase no bottleneck events occurred for the populations surveyed using either of the bottleneck statistics (i.e., Wilcoxon sign-rank test or allele shape distribution).

### Geographic Source

#### Introduction Phase

The minimum F_ST_ and highest *L*_i→s_ values suggested that Serbia was the geographic source of individuals to Croatia and Hungary (Table 3). The combined values suggested that the USA (Iowa-Illinois) was the possible source of WCR to Italy in 2001.

#### Establishment/Spread Phase

Either the Serbian, Croatian or Hungarian populations from the introduction phase were the source of the same populations during the establishment/spread phase populations, including the 2009/10 Pordenone district populations and the 2010 Italian population (Table 3). The 2001 Italian populations were the likely source of the Italian populations sampled in 2009 (Venezia district) (Table 3).

### Isolation by Distance

#### Introduction Phase

**Fig 4:**
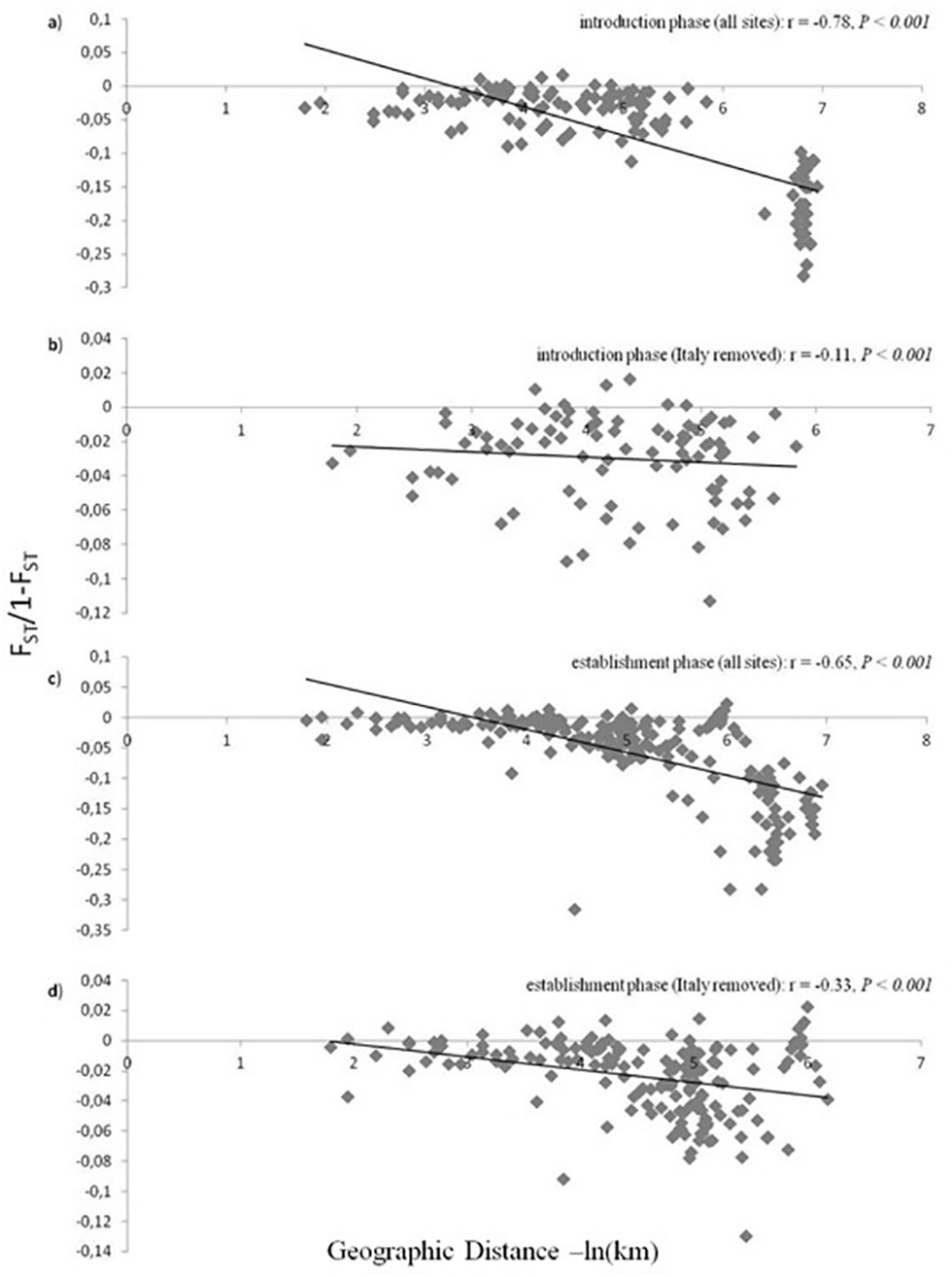
Isolation by distance plots. Genetic distance was plotted as a function of geographic distance for: a) all sites sampled during the introduction phase; b) all sites sampled during the introduction phase excluding the Italian locations; c) all sites sampled during the establishment phase; and d) all locations sampled during the establishment phase excluding the Italian locations. The line of best fit for the data were included for illustration only. r values represent the correlation between geographic and genetic distances matrices assessed using Mantel tests.

**Fig 5:**
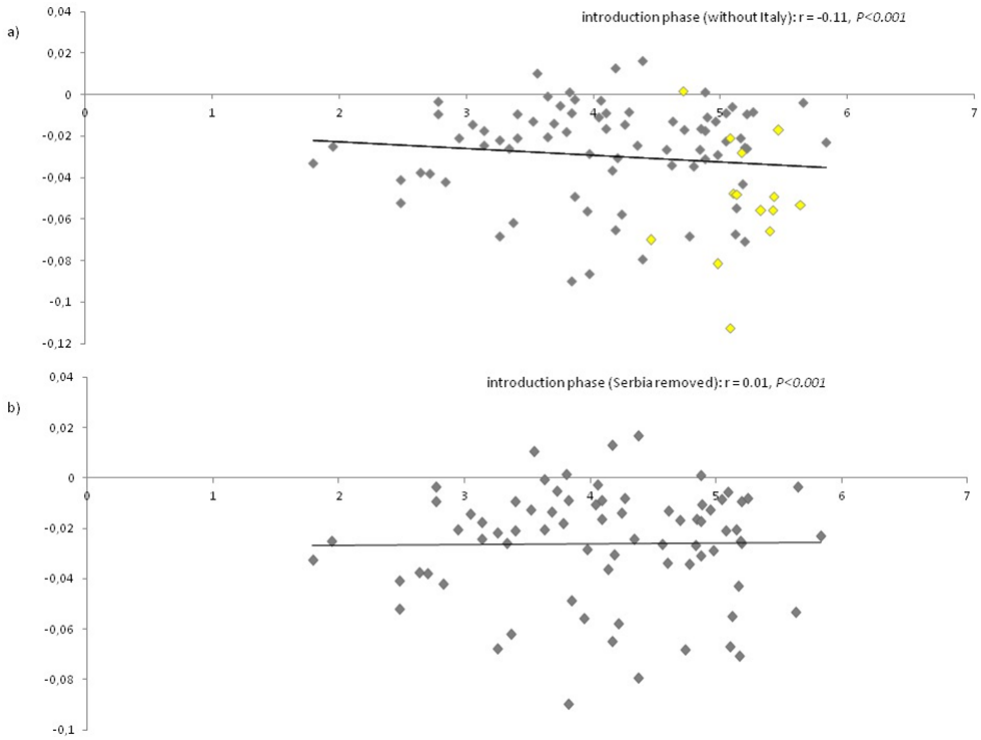
Isolation by distance plots. Genetic distance was plotted as a function of geographic distance for: a) all sites sampled during the introduction phase excluding the Italian locations with identification of Serbia samples (in yellow); b) all sites sampled during the introduction phase excluding the Italian locations and identified source population from Serbia. The line of best fit for the data were included for illustration only. r values represent the correlation between geographic and genetic distances matrices assessed using Mantel tests.

A Mantel test of isolation by distance revealed a significant relationship between Slatkin’s (1995) linearized F_ST_ and the ln of geographic distance in kilometres for populations sampled during the introduction phase (r = -0.78, *P* < 0.001) (Fig 4A). When the most distant Italian populations were removed from the analyses, a low but significant isolation by distance was still found (r = -0.11, *P* < 0.001) (Fig 4B). Serbia was identified as the source population for the first introduced European populations, and was included in analyses to examine gene flow under this scenario (Fig 5A). After removing Serbia from analyses, no isolation by distance was found (r = 0.01, *P* < 0.001) (Fig 5B).

#### Establishment/Spread Phase

For the same populations sampled during the establishment phase a significant isolation by distance pattern of gene flow was found (r = -0.65, *P* < 0.001) (Fig 4C). Again when all Italian populations were removed from the analysis on the establishment phase, a low to moderate, but significant isolation by distance pattern was found (r = -0.33, *P* < 0.001) (Fig 4D).

## Discussion

In this study we widened the scope of our genetic survey of WCR to include countries neighbouring Croatia, where some of the first WCR introductions into Europe occurred. Our results are critical to further understanding the population dynamics of the WCR during the major phases of its European invasion over a 15 year period.

During the introduction phase the number of alleles was low (19–27; 45%) in southern Europe compared to possible native populations from North America (Iowa or Illinois) that we also genotyped. Within a relatively short period of time and coinciding with what we have deemed to be the establishment/spread phase of the invasion, the number of alleles present in southern Europe approximately doubled. Of all known WCR alleles [17,21,22] 84% were now found in southern Europe, 14 years after their first introduction. During the establishment/spread phase it was revealed that Croatia had almost double the number of alleles than the other countries genotyped. However, this may be an artefact of the intensive temporal and spatial sampling program [42] we carried out in Croatia in this study and in Lemic et al. [18]. In comparison, its neighbouring countries were not as intensively sampled by us or others [15,4,19]. To rectify the issue of unequal sample size across populations we calculated rarefied allelic counts for all populations in all sampling periods. The results of the rarefied counts confirm our original findings that during the introduction phase allelic richness was low but consistent in all southern European populations. Conversely, during the establishment/spread phase values were higher for all southern European populations and Croatian populations had significantly higher allelic diversities than any other European populations investigated for that phase.

Our analyses revealed previously undiscovered alleles during the introduction and establishment/spread phases. That is, in the introduction phase two unique alleles were found while during the establishment/spread phase nine previously unrecorded alleles were found. The large number of unique alleles that we found possibly reflects multiple and on-going invasions into southern Europe from different geographic locations both from within Europe and the USA.

Founder events during range expansions, agricultural management using crop rotation, and selection due to insecticide applications are known to affect how genetic diversity in insect species is partitioned (e.g., Colorado potato beetle; [43]). And in this study one of these factors may explain the reason why low levels of genetic variation were found during the introduction phase of the WCR invasion in southern Europe. Alternatively, it is possible that, as a result of multiple introductions and admixture, there was an increase in genetic diversity which was reported as giving rise to novel genotypes in invasive populations [44,45]. Admixture combines genetic variation from multiple genetically differentiated source populations to increase genetic variation within introduced populations [44]. The results of our study, because of their temporal and spatial nature, provide information about the way in which this increase may have occurred. It was possible to observe a loss of genetic variation during the introduction phase followed by a sharp increase in genetic variation during the establishment/spread phase, most likely a result of multiple introductions and admixture [3,18,19]. Admixture is thought to have a positive impact on invasion, through the generation of novel genotypes, an increase in intra-population genetic variation and heterosis, in which admixed individual’s fitness exceeds that of the parental population [19].

Microsatellite based F_ST_ estimates of populations sampled in Croatia, Serbia, and Hungary showed an absence of genetic structure. This indicates that these populations are genetically similar and exist as a single panmictic population over a large geographic area. These results were comparable to those found previously in studies examining the genetic structure of the WCR in central and south eastern Europe (CSE) [2,4,18]. Conversely, our findings show considerable genetic differentiation between Italy and other southern European and USA population comparisons during both invasion phases investigated. Of particular note is the large genetic differentiation (F_ST_ = 0.22) found between geographically close (<50km) Italian pairwise population comparisons during the establishment/spread phase (Venezia district vs. Pordenone district), suggesting possible multiple introductions (from the USA) and admixture events. In the well-known population genetics study by Ciosi et al. [3] the authors showed that WCR entered Europe through five introductions mostly from the USA and central-southern Europe (CSE). In our study we have identified possibly another introduction from the USA into the Venezia district in the period before 2009. While Ciosi et al. [3] identified CSE as the possible source of Venezia district populations our results suggest that this region’s source of WCR was the USA. These unusually high F_ST_ estimates for populations in close proximity, in addition to the STRUCTURE results indicate a separate introduction from the USA, which may have resulted in the failure of the eradication program conducted in 2002 in the Venezia district [46]. Kim and Sappington [47] made it clear that the eradication of low-level populations is difficult, and reintroductions can set-back eradication programs. Such introductions can result in increasing genetic diversity in invaded regions and lead to the redistribution of genetic variation from the intra- to the inter-population level [3].

Genetic differentiation overall was lower in the introduction phase than in the establishment/spread phase. Intercontinental comparisons were highly differentiated. When comparing populations from the introduction phase with the same populations sampled during the establishment/spread phase moderate to high genetic differentiation was found for all comparisons. We first observed this in Lemic et al. [18] where there was decreased genetic variation in the north westerly direction of the establishment/spread during the 14 year investigation carried out; the exception to this being Italy. In southern Europe, namely Serbia, Hungary and Croatia, integrated pest management was established almost 10 years after its first introduction and may have caused the decrease in genetic variation and increase in differentiation found.

Italian populations sampled during the introduction phase revealed low F_ST_ estimates and at that point in time there was no evidence of genetic differentiation. However, during the establishment/spread phase we analysed three different regions close to the locations first invaded. There we found high genetic differentiation between pairwise population comparisons at relatively short distances of up to only 50 km. According to the STRUCTURE analyses conducted in numerous publications [18,48,49] in this survey STRUCTURE analyses were performed according different invasion phases. During the introduction phase, only a single genetic cluster (*K* = 1) was found in southern Europe (which included WCR from Italy), distinct from the putative native source genetic cluster. During the establishment/spread phase also only one genetic cluster (*K* = 1) existed in this area, however, a single population from Italy (Venezia district) clustered with the USA populations. As already discussed this may indicate a second introduction into Italy from the USA in ca. 2001–2 and is contrary to the findings of Ciosi et al. [3] and Bermond et al. [19] who both show CSE as the most probable source of the WCR in the region. However, to further confirm our findings further genetic analyses should be conducted using mitochondrial DNA markers such as the cytochrome oxidase region I (*COI*) gene. Nevertheless, this region is notable for its unusually high F_ST_ values in its pairwise comparisons with all other populations surveyed in this study.

The control methods practised in southern Europe and elsewhere in Europe focus on crop rotation [50–53]. Crop rotation should be fragmenting populations and forcing them into bottlenecks (see Kim and Sappington [47] for a discussion on this in boll weevils, *Anthomous grandis grandis* Boheman). We only found that bottlenecks occurred in southern Europe during the introduction phase (1990–2001) and did not occur at all during the establishment phase (2002–2009) despite ongoing crop rotation. Contrary to the findings of Bermond et al. [19] deviations from HWE occurred during the introduction phase (Croatian and Italian populations only) and the establishment/spread phase (for all populations). These results suggest that current control practises are not exerting pressure on populations to the extent that they used to (during the introduction phase) or intend to, potentially because of repeated introductions (from their USA ancestral source). This then coupled with admixture events has rendered WCR on the whole unaffected by management practises, and populations are now effectively in migration/genetic drift equilibrium.

A stronger isolation by distance pattern was found during the introduction phase compared to the establishment phase and both were significant. The strength of the isolation by distance pattern depended on whether Italian populations were included. Regardless, of the inclusion of Italy significant patterns do emerge, and suggest that populations may be fragmented, and we assume that this is occurring because of improved control practises. However, the impact it is having on overall genetic diversity is minimal, probably for the reasons discussed directly above related to the finding of the bottleneck results. When Serbia was removed from analyses no isolation by distance occurred, suggesting unrestricted gene flow during the first few years after introduction which initially created the southern European WCR population that then acted as the source for later CSE invasions [3,19].

Our results suggest Serbia as the initial source of WCR to its neighbouring countries. With the exception of the population from Venezia district (Italy), which was possibly a result of an introduction from the USA. Also, introductory populations were the subsequent source of individuals sampled during the establishment/spread phase. These results highlight that introductions of WCR to Europe from the USA are ongoing and must be factored into monitoring procedures, current management and control strategies. Genetic monitoring is an invaluable tool to support on ground pest management practises as it can inform managers and scientists alike as to the efficacy of practises by demonstrating how practises (e.g., crop rotation) impact upon the genetics of the populations being controlled.

## Conclusions

Empirical data and anecdotal evidence suggests that current control practises are working [48,50] in southern Europe but their effectiveness is potentially being undermined by the lack of associated cross-country quarantine measures. This research has shown that the loss of genetic variation experienced during the introduction phase was temporary as only a decade later 83% of the known alleles from the USA were present in southern Europe. Future management strategies must look to incorporating genetic monitoring into their management plans. With genetic diversity in southern Europe approaching parity with ancestral populations in the USA comes the real possibility of resistant alleles entering Europe, potentially resulting in resistance to crop rotation as experienced in the USA. Evidently chemical, cultural and other control practises alone are not the answer and genetic monitoring can be used to enhance integrated strategies for WCR control.

## Supporting Information

S1 Fig a) ΔK as per Evanno et al. (2005) plotted against number of genotype clusters (K) (see text), where *K* = 2 is the best fit of the populations from introduction phase. b) ΔK as per Evanno et al. (2005) plotted against number of genotype clusters (k) (see text), where *K* = 2 is the best fit of the populations from establishment/spread phase. (TIF)

S2 Fig Genetic clustering of all investigated populations from the introduction and establishment/spread phases (1—Croatia 1996; 2 –Croatia 2009, 3—Hungary 1996, 4 –Hungary 2009; 5 –Serbia 1996; 6 –Serbia 2009; 7 –Italy 2001; 8—Italy 2009 [Venezia district]; 9—Italy 2009 [Pordenone district]; 10—Italy 2010; 11 –USA [Arizona 2009, Iowa 2011, Illinois 2011, Nebraska 2011]. Additional plots are presented for *K* = 2 (A); *K* = 3 (B); *K* = 4 (C); *K* = 5 (D); *K* = 6 (E).
(TIF)

S1 Table A comparison of published WCR allele frequencies [3,15,16,19,20] and WCR sampled in Croatia, Hungary, Serbia, Italy and the USA during the introduction (1996–2001) and establishment/spread phases of invasion (2002–2011). Alleles in parentheses are unique to the USA. The six loci comprise the *Diabrotica* microsatellite core-set [15]. Alleles in italics are unique in Croatia. Underlined alleles are found in Croatia and USA but not elsewhere in Europe. Shaded alleles indicate unique alleles to Europe. *n*: number of individuals.
(DOCX)

S2 Table Pairwise estimates of F_ST_ (below diagonal) and approximate geographic distances (km, above diagonal) of WCR sampled in Croatia, Hungary, Serbia, Italy (introduction (I) and establishment/spread (E/S) phases) and populations from the USA (native area). Underlined values were significant after Bonferroni correction for multiple comparisons (*n* = 975). Location names underlined were sampled during the introduction phase and again during the establishment/spread phase. Non-abbreviated location names are listed in Table 1.
(DOCX)
