# Supplementary figures and images for: The Temporal and Spatial Invasion Genetics of the Western Corn Rootworm (Coleoptera: Chrysomelidae) in Southern Europe
Source: PLoS One. 2015 Sep 25;10(9):e0138796. doi: 10.1371/journal.pone.0138796 (PMC4583188; doi:10.1371/journal.pone.0138796)

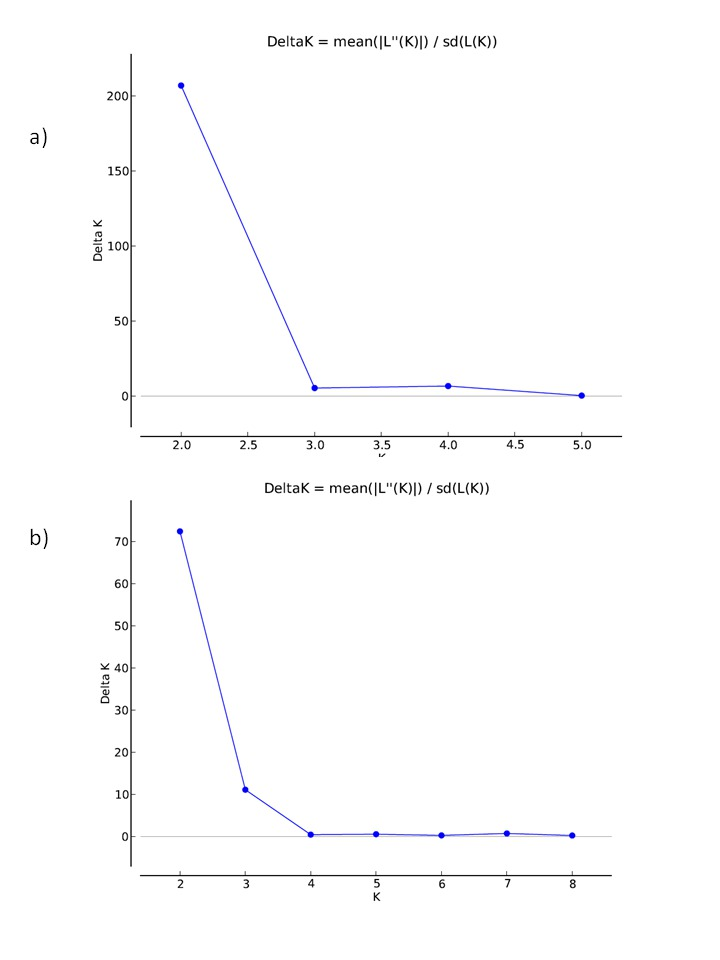

Supplement: S1 Fig — (TIF) [file pone.0138796.s001.tif]

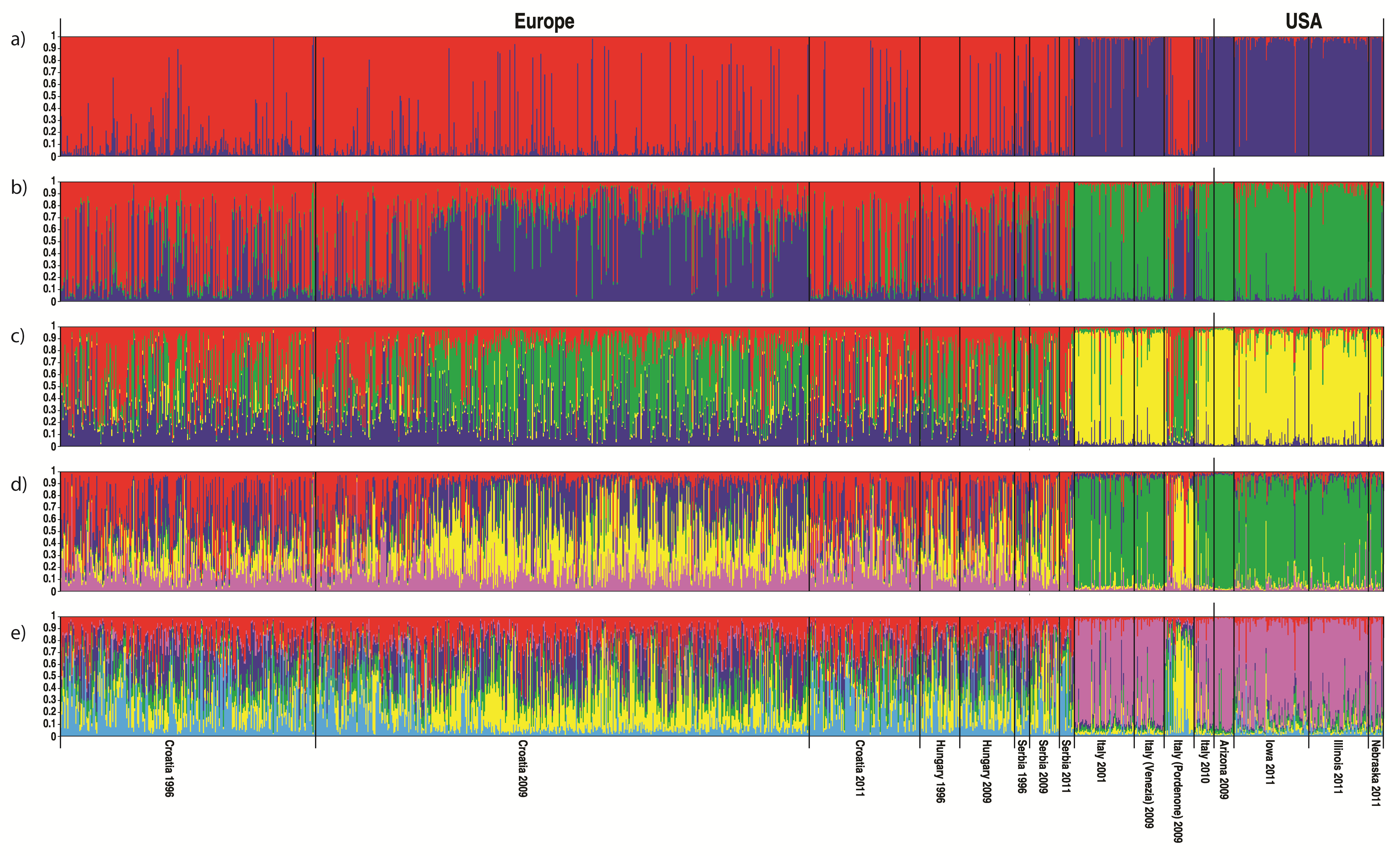

Supplement: S2 Fig — Additional plots are presented for K = 2 (A); K = 3 (B); K = 4 (C); K = 5 (D); K = 6 (E). (TIF) [file pone.0138796.s002.tif]
