# Supplementary material for: The Temporal and Spatial Invasion Genetics of the Western Corn Rootworm (Coleoptera: Chrysomelidae) in Southern Europe
Source: PLoS One. 2015 Sep 25;10(9):e0138796. doi: 10.1371/journal.pone.0138796 (PMC4583188; doi:10.1371/journal.pone.0138796)
Supplement: S1 Table — Alleles in parentheses are unique to the USA. The six loci comprise the Diabrotica microsatellite core-set [15]. Alleles in italics are unique in Croatia. Underlined alleles are found in Croatia and USA but not elsewhere in Europe. Shaded alleles indicate unique alleles to Europe. n: number of individuals. (DOCX) [file pone.0138796.s003.docx]

**S1 Table. A comparison of published WCR allele frequencies [3,15,16,19,20] and WCR sampled in Croatia, Hungary, Serbia, Italy and the USA during the introduction (1996 - 2001) and establishment phases of invasion (2002-2011).** Alleles in parentheses are unique to the USA. The six loci comprise the *Diabrotica* microsatellite core-set [15]. Alleles in italics are unique in Croatia. Underlined alleles are found in Croatia and USA but not elsewhere in Europe. Shaded alleles indicate unique alleles to Europe. *n*: number of individuals.

|  |  | Croatia | | Hungary | | | | Serbia | | Italy | | | USA | |
| --- | --- | --- | --- | --- | --- | --- | --- | --- | --- | --- | --- | --- | --- | --- |
| Locus | Allele | 1996 | 2009 | 2011 | 1996 | 2009 | 1996 | 2009 | 2011 | 2001 | 2009 | 2010 | 2009 | 2011 |
| *n* |  | 256 | 495 | 111 | 40 | 55 | 15 | 30 | 15 | 60 | 30 | 20 | 20 | 150 |
| DVV-D2 | 177 | 0 | 0 | 0.005 | 0 | 0 | 0 | 0 | 0 | 0 | 0 | 0 | 0.075 | 0.010 |
| (dinucleotide) | (179) | 0 | 0 | 0 | 0 | 0 | 0 | 0 | 0 | 0 | 0 | 0 | 0.075 | 0 |
|  | 181 | 0.227 | 0.069 | 0.198 | 0.191 | 0.139 | 0.233 | 0.179 | 0.233 | 0.043 | 0.132 | 0.059 | 0.325 | 0.252 |
|  | 183 | 0.403 | 0.528 | 0.266 | 0.529 | 0.426 | 0.533 | 0.339 | 0.233 | 0.904 | 0.500 | 0.706 | 0.400 | 0.409 |
|  | 185 | 0 | 0.002 | 0.005 | 0 | 0 | 0 | 0 | 0 | 0 | 0 | 0.059 | 0 | 0 |
|  | 187 | 0.156 | 0.248 | 0.203 | 0.118 | 0.222 | 0.133 | 0.089 | 0.200 | 0.011 | 0 | 0 | 0 | 0.037 |
|  | (189) | 0 | 0 | 0 | 0 | 0 | 0 | 0 | 0 | 0 | 0 | 0 | 0 | 0.047 |
|  | 191 | 0.002 | 0.004 | 0 | 0 | 0 | 0 | 0 | 0 | 0 | 0 | 0 | 0 | 0.003 |
|  | 193 | 0 | 0 | 0 | 0 | 0 | 0 | 0 | 0.033 | 0 | 0 | 0 | 0.025 | 0 |
|  | 197 | 0 | 0 | 0.005 | 0 | 0 | 0 | 0 | 0 | 0 | 0 | 0 | 0 | 0 |
|  | 199 | 0 | 0 | 0 | 0 | 0 | 0 | 0 | 0 | 0 | 0 | 0.029 | 0.025 | 0.010 |
|  | 201 | 0.004 | 0.064 | 0.009 | 0.044 | 0.056 | 0 | 0.232 | 0 | 0.043 | 0.283 | 0.088 | 0 | 0.030 |
|  | 203 | 0.203 | 0.081 | 0.302 | 0.118 | 0.157 | 0.100 | 0.054 | 0.267 | 0 | 0.047 | 0 | 0 | 0.107 |
|  | 205 | 0 | 0 | 0.005 | 0 | 0 | 0 | 0.036 | 0.033 | 0 | 0.019 | 0.059 | 0.050 | 0.047 |
|  | 207 | 0 | 0 | 0.005 | 0 | 0 | 0 | 0.071 | 0 | 0 | 0.019 | 0 | 0.025 | 0.047 |
| DVV-T2 | 204 | 0.002 | 0.013 | 0.009 | 0 | 0 | 0 | 0.017 | 0 | 0 | 0.037 | 0.026 | 0.025 | 0.003 |
| (trinucleotide) | 208 | 0 | 0 | 0 | 0 | 0 | 0 | 0 | 0 | 0 | 0.019 | 0 | 0 | 0 |
|  | 210 | 0.291 | 0.171 | 0.167 | 0.375 | 0.245 | 0.467 | 0.467 | 0.167 | 0.284 | 0.176 | 0.421 | 0.150 | 0.299 |
|  | 213 | 0 | 0 | 0.005 | 0 | 0 | 0 | 0 | 0.067 | 0 | 0 | 0 | 0 | 0 |
|  | 216 | 0 | 0 | 0.005 | 0 | 0 | 0 | 0 | 0 | 0 | 0 | 0 | 0.025 | 0 |
|  | 219 | 0 | 0.003 | 0.045 | 0 | 0.009 | 0 | 0 | 0.033 | 0 | 0 | 0 | 0.100 | 0.114 |
|  | 222 | 0.707 | 0.811 | 0.766 | 0.625 | 0.745 | 0.533 | 0.517 | 0.733 | 0.716 | 0.731 | 0.553 | 0.675 | 0.584 |
|  | 225 | 0 | 0.001 | 0 | 0 | 0 | 0 | 0 | 0 | 0 | 0.019 | 0 | 0.025 | 0 |
|  | 226 | 0 | 0 | 0 | 0 | 0 | 0 | 0 | 0 | 0 | 0.019 | 0 | 0 | 0 |
| Dba05 | 216 | 0 | 0 | 0.005 | 0 | 0 | 0 | 0 | 0 | 0 | 0 | 0 | 0 | 0.010 |
| (trinucleotide) | 219 | 0.439 | 0.522 | 0.496 | 0.313 | 0.436 | 0.300 | 0.417 | 0.600 | 0.232 | 0.389 | 0.237 | 0.250 | 0.345 |
|  | 222 | 0.561 | 0.478 | 0.486 | 0.688 | 0.564 | 0.700 | 0.583 | 0.400 | 0.768 | 0.593 | 0.763 | 0.750 | 0.615 |
|  | 224 | 0 | 0 | 0 | 0 | 0 | 0 | 0 | 0 | 0 | 0.019 | 0 | 0 | 0 |
|  | 225 | 0 | 0 | 0 | 0 | 0 | 0 | 0 | 0 | 0 | 0 | 0 | 0 | 0.030 |
|  | 227 | 0 | 0 | 0.005 | 0 | 0 | 0 | 0 | 0 | 0 | 0 | 0 | 0 | 0 |
|  | 230 | 0 | 0 | 0.005 | 0 | 0 | 0 | 0 | 0 | 0 | 0 | 0 | 0 | 0 |
|  | 237 | 0 | 0 | 0.005 | 0 | 0 | 0 | 0 | 0 | 0 | 0 | 0 | 0 | 0 |
| DVV-D4 | 211 | 0 | 0 | 0.005 | 0 | 0 | 0 | 0 | 0.033 | 0 | 0 | 0 | 0 | 0 |
| (dinucleotide) | 219 | 0.002 | 0.001 | 0 | 0 | 0 | 0 | 0 | 0 | 0 | 0 | 0 | 0.133 | 0.153 |
|  | 221 | 0 | 0 | 0.005 | 0 | 0 | 0 | 0 | 0 | 0 | 0 | 0 | 0 | 0 |
|  | 223 | 0.190 | 0.400 | 0.419 | 0.400 | 0.415 | 0.107 | 0.450 | 0.533 | 0.008 | 0.182 | 0.100 | 0 | 0.143 |
|  | 225 | 0.553 | 0.463 | 0.414 | 0.438 | 0.443 | 0.679 | 0.450 | 0.267 | 0.602 | 0.691 | 0.700 | 0.333 | 0.500 |
|  | 227 | 0 | 0.003 | 0 | 0 | 0 | 0 | 0 | 0.033 | 0.008 | 0.036 | 0 | 0.033 | 0.070 |
|  | 229 | 0 | 0.001 | 0 | 0 | 0 | 0 | 0 | 0 | 0 | 0.009 | 0 | 0.067 | 0.003 |
|  | 231 | 0.251 | 0.128 | 0.158 | 0.163 | 0.132 | 0.214 | 0.100 | 0.100 | 0.373 | 0.082 | 0.200 | 0.133 | 0.070 |
|  | 233 | 0 | 0.002 | 0 | 0 | 0 | 0 | 0 | 0.033 | 0.008 | 0 | 0 | 0.300 | 0.023 |
|  | 235 | 0.004 | 0.002 | 0 | 0 | 0 | 0 | 0 | 0 | 0 | 0 | 0 | 0 | 0.013 |
|  | (237) | 0 | 0 | 0 | 0 | 0 | 0 | 0 | 0 | 0 | 0 | 0 | 0 | 0.007 |
|  | (239) | 0 | 0 | 0 | 0 | 0 | 0 | 0 | 0 | 0 | 0 | 0 | 0 | 0.017 |
| DVV-D8 | 208 | 0 | 0 | 0 | 0 | 0 | 0 | 0 | 0 | 0 | 0.009 | 0 | 0 | 0 |
| (dinucleotide) | 212 | 0 | 0 | 0.005 | 0 | 0 | 0 | 0 | 0 | 0 | 0 | 0 | 0.050 | 0.054 |
|  | (214) | 0 | 0 | 0 | 0 | 0 | 0 | 0 | 0 | 0 | 0 | 0 | 0.075 | 0.003 |
|  | 216 | 0 | 0 | 0.009 | 0 | 0 | 0.038 | 0.017 | 0 | 0.071 | 0.155 | 0.150 | 0.125 | 0.104 |
|  | 218 | 0.347 | 0.248 | 0.288 | 0.213 | 0.274 | 0.231 | 0.183 | 0.267 | 0.009 | 0 | 0 | 0.025 | 0.245 |
|  | 220 | 0.002 | 0.006 | 0.009 | 0 | 0.009 | 0 | 0 | 0 | 0.009 | 0 | 0 | 0.200 | 0.074 |
|  | 222 | 0 | 0.003 | 0.005 | 0 | 0 | 0 | 0 | 0 | 0.027 | 0 | 0.025 | 0 | 0.070 |
|  | 224 | 0 | 0 | 0 | 0 | 0 | 0 | 0 | 0 | 0.241 | 0 | 0.075 | 0 | 0.064 |
|  | 226 | 0 | 0.002 | 0.005 | 0 | 0 | 0 | 0 | 0 | 0.009 | 0 | 0 | 0.050 | 0.030 |
|  | 228 | 0 | 0.010 | 0 | 0 | 0 | 0 | 0.017 | 0 | 0 | 0 | 0 | 0.175 | 0.003 |
|  | (230) | 0 | 0 | 0 | 0 | 0 | 0 | 0 | 0 | 0 | 0 | 0 | 0.075 | 0.007 |
|  | (232) | 0 | 0 | 0 | 0 | 0 | 0 | 0 | 0 | 0 | 0 | 0 | 0.050 | 0.020 |
|  | 234 | 0 | 0.001 | 0 | 0 | 0 | 0 | 0 | 0 | 0 | 0 | 0 | 0 | 0.003 |
|  | 236 | 0 | 0.001 | 0.005 | 0 | 0 | 0 | 0 | 0 | 0 | 0 | 0 | 0.025 | 0.007 |
|  | (238) | 0 | 0 | 0 | 0 | 0 | 0 | 0 | 0 | 0 | 0 | 0 | 0.075 | 0.003 |
|  | 240 | 0 | 0.001 | 0 | 0 | 0 | 0 | 0 | 0 | 0 | 0 | 0 | 0 | 0.013 |
|  | 242 | 0.004 | 0.003 | 0 | 0 | 0 | 0.038 | 0 | 0.067 | 0.286 | 0.109 | 0.300 | 0 | 0.074 |
|  | 244 | 0.061 | 0.090 | 0.126 | 0.050 | 0.038 | 0.077 | 0.083 | 0.067 | 0 | 0 | 0.025 | 0 | 0.044 |
|  | 246 | 0.414 | 0.498 | 0.423 | 0.688 | 0.557 | 0.462 | 0.567 | 0.467 | 0.063 | 0.382 | 0.200 | 0 | 0.094 |
|  | 248 | 0.161 | 0.135 | 0.126 | 0.050 | 0.094 | 0.115 | 0.133 | 0.133 | 0.027 | 0.100 | 0.025 | 0 | 0.027 |
|  | 250 | 0 | 0.001 | 0 | 0 | 0.019 | 0.038 | 0 | 0 | 0.250 | 0.227 | 0.200 | 0.025 | 0.050 |
|  | (252) | 0 | 0 | 0 | 0 | 0 | 0 | 0 | 0 | 0 | 0 | 0 | 0.025 | 0.010 |
| Dba07 | 215 | 0.218 | 0.191 | 0.266 | 0.238 | 0.139 | 0.308 | 0.233 | 0.267 | 0.465 | 0.318 | 0.475 | 0.050 | 0.157 |
| (tetranucleotide) | 217 | 0.002 | 0 | 0 | 0 | 0.009 | 0 | 0 | 0 | 0 | 0 | 0 | 0 | 0 |
|  | 219 | 0 | 0.018 | 0 | 0 | 0.009 | 0 | 0.033 | 0 | 0.018 | 0.055 | 0 | 0.475 | 0.420 |
|  | 223 | 0 | 0.006 | 0 | 0 | 0 | 0 | 0 | 0 | 0 | 0 | 0 | 0.275 | 0.010 |
|  | (227) | 0 | 0 | 0 | 0 | 0 | 0 | 0 | 0 | 0 | 0 | 0 | 0.050 | 0.013 |
|  | (229) | 0 | 0 | 0 | 0 | 0 | 0 | 0 | 0 | 0 | 0 | 0 | 0 | 0.007 |
|  | 231 | 0 | 0 | 0.005 | 0 | 0 | 0 | 0 | 0 | 0 | 0 | 0 | 0 | 0 |
|  | 235 | 0.523 | 0.522 | 0.486 | 0.413 | 0.639 | 0.346 | 0.517 | 0.500 | 0.491 | 0.364 | 0.475 | 0.150 | 0.383 |
|  | 237 | 0.006 | 0.001 | 0 | 0 | 0.009 | 0 | 0 | 0 | 0.018 | 0.009 | 0 | 0 | 0 |
|  | 239 | 0.250 | 0.262 | 0.243 | 0.350 | 0.194 | 0.346 | 0.217 | 0.233 | 0.009 | 0.245 | 0.050 | 0 | 0.010 |
|  | (241) | 0 | 0 | 0 | 0 | 0 | 0 | 0 | 0 | 0 | 0.009 | 0 | 0 | 0 |
