# Supplementary material for: The Temporal and Spatial Invasion Genetics of the Western Corn Rootworm (Coleoptera: Chrysomelidae) in Southern Europe
Source: PLoS One. 2015 Sep 25;10(9):e0138796. doi: 10.1371/journal.pone.0138796 (PMC4583188; doi:10.1371/journal.pone.0138796)
Supplement: S2 Table — Underlined values were significant after Bonferroni correction for multiple comparisons (n = 975). Location names underlined were sampled during the introduction phase and again during the establishment/spread phase. Non-abbreviated location names are listed in Table 1. (DOCX) [file pone.0138796.s004.docx]

**S2 Table. Pairwise estimates of F_ST_ (below diagonal) and approximate geographic distances (km, above diagonal) of WCR sampled in Croatia, Hungary, Serbia, Italy (introduction (I) and establishment/spread (E/S) phases) and populations from the USA (native area).** Underlined values were significant after Bonferroni correction for multiple comparisons (*n* = 975). Location names underlined were sampled during the introduction phase and again during the establishment/spread phase. Non-abbreviated location names are listed in Table 1.

| **Croatia** | | | | | | | | | | | | | | | | | | | | | | | | | | | | **Hungary** | | | | **Serbia** | | **Italy** | | | | | |
| --- | --- | --- | --- | --- | --- | --- | --- | --- | --- | --- | --- | --- | --- | --- | --- | --- | --- | --- | --- | --- | --- | --- | --- | --- | --- | --- | --- | --- | --- | --- | --- | --- | --- | --- | --- | --- | --- | --- | --- |
|  | **I** | | | | | | | | | | | **E/S** | | | | | | | | | | | | | | | | **I** | | **E/S** | | **I** | **E/S** | **I** | | | **E/S** | | |
| **Pop** | **Bos** | **Dre** | **Nus** | **Oto** | **Rac** | **Sol** | **Mik** | **Tov** | **Vrb** | **Vrp** | **P.M.** | **Ban** | **DM** | **Dol** | **Bos** | **Dre** | **Nus** | **Oto** | **Rac** | **Sol** | **Mik** | **Tov** | **Vrb** | **Vrp** | **Ogu** | **Gun** | **Poz** | **Moh** | **Pec** | **Moh** | **Pec** | **Sur** | **Sur** | **Apg** | **Rim** | **Vai** | **Ven** | **Por** | **Laz** |
| **Bos**  **1996** | - | 19 | 38 | 15 | 26 | 21 | 30 | 34 | 16 | 35 | 155 | 136 | 102 | 121 | - | - | - | - | - | - | - | - | - | - | 359 | 23 | 128 | 160 | 204 | - | - | 145 | - | 915 | 887 | 865 | 632 | 589 | 919 |
| **Dre**  **1996** | 0.02 | - | 65 | 30 | 12 | 12 | 68 | 47 | 7 | 70 | 177 | 158 | 124 | 143 | - | - | - | - | - | - | - | - | - | - | 381 | 9 | 150 | 182 | 226 | - | - | 133 | - | 973 | 909 | 887 | 654 | 611 | 942 |
| **Nus**  **1996** | 0 | 0.06 | - | 26 | 77 | 63 | 28 | 40 | 57 | 45 | 102 | 157 | 123 | 112 | - | - | - | - | - | - | - | - | - | - | 380 | 62 | 149 | 101 | 145 | - | - | 166 | - | 935 | 908 | 886 | 653 | 610 | 940 |
| **Oto**  **1996** | 0.04 | 0.02 | 0.06 | - | 42 | 29 | 46 | 23 | 23 | 38 | 131 | 154 | 120 | 138 | - | - | - | - | - | - | - | - | - | - | 377 | 59 | 146 | 120 | 164 | - | - | 131 | - | 932 | 905 | 883 | 650 | 607 | 937 |
| **Rac**  **1996** | 0.02 | 0.04 | 0.02 | 0.01 | - | 14 | 80 | 65 | 16 | 80 | 182 | 167 | 133 | 152 | - | - | - | - | - | - | - | - | - | - | 393 | 12 | 159 | 191 | 235 | - | - | 142 | - | 946 | 918 | 896 | 663 | 620 | 950 |
| **Sol**  **1996** | 0.01 | 0.05 | 0.04 | 0.06 | 0.04 | - | 66 | 44 | 6 | 53 | 168 | 156 | 122 | 141 | - | - | - | - | - | - | - | - | - | - | 380 | 15 | 148 | 180 | 224 | - | - | 131 | - | 935 | 907 | 885 | 652 | 609 | 940 |
| **Mik**  **1996** | 0.01 | 0.05 | 0.03 | 0.08 | 0.07 | 0.03 | - | 52 | 60 | 17 | 97 | 98 | 94 | 72 | - | - | - | - | - | - | - | - | - | - | 350 | 75 | 90 | 116 | 160 | - | - | 179 | - | 907 | 879 | 614 | 624 | 581 | 742 |
| **Tov**  **1996** | 0.01 | 0.05 | 0.01 | 0.02 | 0 | 0.02 | 0.05 | - | 46 | 58 | 127 | 187 | 153 | 136 | - | - | - | - | - | - | - | - | - | - | 403 | 70 | 179 | 125 | 169 | - | - | 111 | - | 965 | 938 | 916 | 683 | 640 | 970 |
| **Vrb**  **1996** | 0 | 0.02 | 0.01 | 0.02 | 0.01 | 0.03 | 0.02 | 0.01 | - | 60 | 163 | 151 | 117 | 135 | - | - | - | - | - | - | - | - | - | - | 374 | 16 | 142 | 175 | 219 | - | - | 126 | - | 929 | 902 | 880 | 647 | 604 | 934 |
| **Vrp**  **1996** | 0.00 | 0.01 | 0.00 | 0.02 | 0 | 0.03 | 0.04 | 0 | 0.01 | - | 72 | 85 | 69 | 68 | - | - | - | - | - | - | - | - | - | - | 249 | 73 | 76 | 111 | 155 | - | - | 174 | - | 882 | 854 | 832 | 599 | 556 | 886 |
| **PM**  **1996** | 0.01 | 0.04 | 0.01 | 0.03 | 0.01 | 0.05 | 0.03 | 0.02 | 0.01 | 0.01 | - | 82 | 115 | 32 | - | - | - | - | - | - | - | - | - | - | 309 | 382 | 95 | 86 | 53 | - | - | 279 | - | 867 | 839 | 817 | 601 | 541 | 871 |
| **Ban**  **2009** | 0 | 0.05 | 0 | 0.08 | 0.05 | 0.06 | 0.02 | 0.04 | 0.02 | 0 | 0.01 | - | 45 | 10 | - | - | - | - | - | - | - | - | - | - | 282 | 158 | 7 | 155 | 125 | - | - | 259 | - | 837 | 659 | 626 | 572 | 511 | 669 |
| **DM**  **2009** | 0.02 | 0.06 | 0.01 | 0.09 | 0.04 | 0.08 | 0.04 | 0.05 | 0.03 | 0 | 0.03 | 0 | - | 68 | - | - | - | - | - | - | - | - | - | - | 136 | 91 | 37 | 205 | 161 | - | - | 448 | - | 850 | 665 | 631 | 416 | 355 | 536 |
| **Dol**  **2009** | 0.00 | 0.04 | 0.00 | 0.05 | 0.01 | 0.05 | 0.03 | 0.02 | 0.01 | 0 | 0.01 | 0 | 0 | - | - | - | - | - | - | - | - | - | - | - | 328 | 140 | 49 | 122 | 87 | - | - | 241 | - | 883 | 855 | 649 | 617 | 557 | 888 |
| **Bos**  **2009** | 0.05 | 0.07 | 0.06 | 0.05 | 0.04 | 0.09 | 0.10 | 0.04 | 0.05 | 0.04 | 0.07 | 0.06 | 0.05 | 0.03 | - | - | - | - | - | - | - | - | - | - | - | - | - | - | - | - | - | - | - | - | - | - | - | - | - |
| **Dre**  **2009** | 0.03 | 0.08 | 0.03 | 0.07 | 0.04 | 0.08 | 0.06 | 0.03 | 0.05 | 0.03 | 0.02 | 0.02 | 0.02 | 0.01 | 0.02 | - | - | - | - | - | - | - | - | - | - | - | - | - | - | - | - | - | - | - | - | - | - | - | - |
| **Nus**  **2009** | 0.04 | 0.09 | 0.05 | 0.06 | 0.05 | 0.07 | 0.08 | 0.03 | 0.06 | 0.05 | 0.05 | 0.06 | 0.06 | 0.03 | 0.01 | 0 | - | - | - | - | - | - | - | - | - | - | - | - | - | - | - | - | - | - | - | - | - | - | - |
| **Oto**  **2009** | 0.03 | 0.07 | 0.04 | 0.06 | 0.04 | 0.06 | 0.09 | 0.03 | 0.06 | 0.02 | 0.06 | 0.04 | 0.05 | 0.02 | 0.01 | 0.01 | 0.01 | - | - | - | - | - | - | - | - | - | - | - | - | - | - | - | - | - | - | - | - | - | - |
| **Rac**  **2009** | 0.05 | 0.12 | 0.04 | 0.09 | 0.07 | 0.08 | 0.07 | 0.04 | 0.06 | 0.07 | 0.06 | 0.05 | 0.06 | 0.04 | 0.01 | 0 | 0.01 | 0.02 | - | - | - | - | - | - | - | - | - | - | - | - | - | - | - | - | - | - | - | - | - |
| **Sol**  **2009** | 0.04 | 0.09 | 0.04 | 0.08 | 0.05 | 0.09 | 0.09 | 0.05 | 0.06 | 0.04 | 0.05 | 0.04 | 0.03 | 0.01 | 0.01 | 0 | 0.01 | 0.01 | 0.01 | - | - | - | - | - | - | - | - | - | - | - | - | - | - | - | - | - | - | - | - |
| **Mik**  **2009** | 0.04 | 0.09 | 0.03 | 0.08 | 0.03 | 0.08 | 0.09 | 0.03 | 0.05 | 0.02 | 0.07 | 0.04 | 0.03 | 0.02 | 0.01 | 0.02 | 0.02 | 0.01 | 0.02 | 0 | - | - | - | - | - | - | - | - | - | - | - | - | - | - | - | - | - | - | - |
| **Tov**  **2009** | 0.04 | 0.08 | 0.04 | 0.04 | 0.02 | 0.08 | 0.09 | 0.02 | 0.04 | 0.02 | 0.04 | 0.05 | 0.04 | 0.02 | 0 | 0.01 | 0 | 0 | 0 | 0 | 0 | - | - | - | - | - | - | - | - | - | - | - | - | - | - | - | - | - | - |
| **Vrb**  **2009** | 0.02 | 0.05 | 0.02 | 0.03 | 0.01 | 0.04 | 0.06 | 0.00 | 0.03 | 0.00 | 0.02 | 0.03 | 0.03 | 0.01 | 0 | 0 | 0 | 0 | 0 | 0 | 0.01 | 0 | - | - | - | - | - | - | - | - | - | - | - | - | - | - | - | - | - |
| **Vrp**  **2009** | 0.03 | 0.07 | 0.03 | 0.05 | 0.04 | 0.06 | 0.08 | 0.02 | 0.04 | 0.03 | 0.05 | 0.04 | 0.05 | 0.03 | 0.01 | 0.01 | 0 | 0 | 0.02 | 0.01 | 0.02 | 0 | 0 | - | - | - | - | - | - | - | - | - | - | - | - | - | - | - | - |
| **Ogu**  **2009** | 0.04 | 0.09 | 0.05 | 0.04 | 0.01 | 0.08 | 0.09 | 0.02 | 0.04 | 0.03 | 0.04 | 0.06 | 0.04 | 0.02 | 0 | 0 | 0 | 0.01 | 0 | 0 | 0.01 | 0 | 0 | 0.01 | - | - | - | 432 | 340 | - | - | 483 | - | 599 | 571 | 549 | 317 | 273 | 604 |
| **Gun**  **2009** | 0.04 | 0.07 | 0.04 | 0.05 | 0.03 | 0.08 | 0.09 | 0.03 | 0.05 | 0.02 | 0.05 | 0.04 | 0.03 | 0.02 | 0 | 0.01 | 0.02 | 0 | 0.02 | 0 | 0 | 0 | 0 | 0.01 | 0 | - | 151 | 183 | 227 | - | - | 142 | - | 938 | 910 | 888 | 656 | 612 | 934 |
| **Poz**  **2009** | 0.01 | 0.03 | 0.03 | 0.03 | 0.01 | 0.04 | 0.06 | 0.01 | 0.02 | 0.01 | 0.04 | 0.04 | 0.04 | 0.01 | 0.01 | 0.02 | 0.01 | 0.01 | 0.03 | 0.02 | 0.01 | 0.01 | 0.00 | 0.01 | 0.01 | 0.01 | - | 142 | 119 | - | - | 250 | - | 830 | 803 | 781 | 548 | 505 | 835 |
| **Moh**  **2096** | 0.02 | 0.03 | 0.03 | 0.03 | 0.01 | 0.02 | 0.06 | 0.02 | 0.03 | 0 | 0.07 | 0.04 | 0.04 | 0.02 | 0.03 | 0.06 | 0.06 | 0.03 | 0.06 | 0.05 | 0.04 | 0.04 | 0.02 | 0.05 | 0.03 | 0.03 | 0.01 | - | 47 | - | - | 284 | - | 967 | 939 | 917 | 658 | 641 | 972 |
| **Pec**  **2096** | 0.05 | 0.05 | 0.08 | 0.05 | 0.02 | 0.05 | 0.10 | 0.05 | 0.06 | 0.02 | 0.08 | 0.07 | 0.05 | 0.04 | 0.03 | 0.06 | 0.06 | 0.05 | 0.08 | 0.06 | 0.05 | 0.05 | 0.03 | 0.07 | 0.03 | 0.03 | 0.02 | 0.00 | - | - | - | 341 | - | 923 | 896 | 874 | 641 | 598 | 928 |
| **Moh**  **2009** | 0.02 | 0.09 | 0.01 | 0.08 | 0.03 | 0.08 | 0.06 | 0.03 | 0.04 | 0.01 | 0.02 | 0.01 | 0.01 | 0.00 | 0.03 | 0.00 | 0.03 | 0.03 | 0.03 | 0.01 | 0.01 | 0.02 | 0.01 | 0.03 | 0.02 | 0.03 | 0.03 | 0.06 | 0.07 | - | - | - | - | - | - | - | - | - | - |
| **Pec**  **2009** | 0.04 | 0.02 | 0.07 | 0.06 | 0.04 | 0.06 | 0.08 | 0.06 | 0.05 | 0.01 | 0.08 | 0.06 | 0.05 | 0.04 | 0.05 | 0.07 | 0.07 | 0.05 | 0.11 | 0.06 | 0.06 | 0.06 | 0.04 | 0.06 | 0.07 | 0.04 | 0.02 | 0.02 | 0.02 | 0.08 | - | - | - | - | - | - | - | - | - |
| **Sur**  **1996** | 0.03 | 0.01 | 0.06 | 0.00 | 0.01 | 0.02 | 0.07 | 0.02 | 0.03 | 0.02 | 0.05 | 0.08 | 0.09 | 0.06 | 0.06 | 0.08 | 0.07 | 0.06 | 0.09 | 0.10 | 0.09 | 0.06 | 0.04 | 0.06 | 0.05 | 0.07 | 0.02 | 0.00 | 0.02 | 0.10 | 0.04 | - | - | 1038 | 1010 | 988 | 756 | 712 | 1043 |
| **Sur**  **2009** | 0.04 | 0.03 | 0.06 | 0.04 | 0.02 | 0.06 | 0.08 | 0.04 | 0.03 | 0.02 | 0.05 | 0.05 | 0.04 | 0.03 | 0.04 | 0.05 | 0.05 | 0.05 | 0.07 | 0.04 | 0.04 | 0.04 | 0.04 | 0.06 | 0.04 | 0.03 | 0.02 | 0.01 | 0.01 | 0.06 | 0.01 | 0.03 | - | - | - | - | - | - | - |
| **Apg**  **2001** | 0.18 | 0.19 | 0.21 | 0.13 | 0.13 | 0.16 | 0.22 | 0.13 | 0.17 | 0.18 | 0.18 | 0.25 | 0.25 | 0.20 | 0.20 | 0.20 | 0.16 | 0.19 | 0.22 | 0.21 | 0.20 | 0.18 | 0.16 | 0.17 | 0.17 | 0.19 | 0.15 | 0.19 | 0.18 | 0.22 | 0.19 | 0.13 | 0.18 | - | 41 | 83 | 292 | 371 | 10 |
| **Rim**  **2001** | 0.15 | 0.18 | 0.18 | 0.12 | 0.13 | 0.12 | 0.19 | 0.11 | 0.16 | 0.16 | 0.17 | 0.23 | 0.24 | 0.19 | 0.18 | 0.19 | 0.14 | 0.17 | 0.19 | 0.20 | 0.19 | 0.17 | 0.14 | 0.15 | 0.15 | 0.18 | 0.13 | 0.16 | 0.16 | 0.21 | 0.18 | 0.10 | 0.17 | 0.002 | - | 43 | 20 | 344 | 47 |
| **Vai**  **2001** | 0.12 | 0.15 | 0.15 | 0.12 | 0.11 | 0.09 | 0.16 | 0.10 | 0.15 | 0.12 | 0.14 | 0.18 | 0.20 | 0.15 | 0.18 | 0.16 | 0.12 | 0.14 | 0.17 | 0.17 | 0.16 | 0.16 | 0.12 | 0.13 | 0.15 | 0.16 | 0.12 | 0.15 | 0.16 | 0.18 | 0.16 | 0.10 | 0.16 | 0.03 | 0.01 | - | 250 | 321 | 96 |
| **Ven**  **2009** | 0.16 | 0.18 | 0.18 | 0.12 | 0.14 | 0.15 | 0.18 | 0.13 | 0.16 | 0.17 | 0.15 | 0.22 | 0.22 | 0.18 | 0.17 | 0.17 | 0.13 | 0.17 | 0.16 | 0.18 | 0.18 | 0.15 | 0.14 | 0.15 | 0.14 | 0.17 | 0.14 | 0.18 | 0.17 | 0.19 | 0.19 | 0.11 | 0.16 | 0.05 | 0.03 | 0.05 | - | 88 | 306 |
| **Por**  **2009** | 0.09 | 0.11 | 0.09 | 0.12 | 0.13 | 0.11 | 0.13 | 0.11 | 0.12 | 0.09 | 0.13 | 0.08 | 0.09 | 0.09 | 0.10 | 0.10 | 0.12 | 0.09 | 0.09 | 0.09 | 0.10 | 0.11 | 0.08 | 0.11 | 0.12 | 0.08 | 0.09 | 0.06 | 0.08 | 0.10 | 0.11 | 0.10 | 0.07 | 0.29 | 0.25 | 0.21 | 0.24 | - | 378 |
| **Laz**  **2001** | 0.11 | 0.11 | 0.15 | 0.07 | 0.09 | 0.09 | 0.14 | 0.09 | 0.11 | 0.11 | 0.12 | 0.17 | 0.18 | 0.13 | 0.13 | 0.14 | 0.11 | 0.13 | 0.15 | 0.15 | 0.14 | 0.13 | 0.11 | 0.12 | 0.11 | 0.13 | 0.09 | 0.11 | 0.10 | 0.16 | 0.11 | 0.04 | 0.10 | 0.03 | 0.01 | 0.02 | 0.02 | 0.18 | - |
| **AZ**  **Will** | 0.15 | 0.16 | 0.16 | 0.16 | 0.15 | 0.13 | 0.17 | 0.15 | 0.16 | 0.15 | 0.15 | 0.18 | 0.18 | 0.16 | 0.20 | 0.18 | 0.17 | 0.18 | 0.17 | 0.19 | 0.20 | 0.19 | 0.15 | 0.16 | 0.17 | 0.18 | 0.16 | 0.15 | 0.18 | 0.20 | 0.17 | 0.13 | 0.16 | 0.20 | 0.17 | 0.14 | 0.16 | 0.22 | 0.15 |
| **IA**  **Nas** | 0.10 | 0.10 | 0.13 | 0.11 | 0.10 | 0.10 | 0.11 | 0.11 | 0.10 | 0.10 | 0.11 | 0.14 | 0.14 | 0.11 | 0.14 | 0.15 | 0.14 | 0.13 | 0.15 | 0.15 | 0.15 | 0.15 | 0.14 | 0.12 | 0.13 | 0.13 | 0.13 | 0.11 | 0.13 | 0.16 | 0.10 | 0.08 | 0.10 | 0.16 | 0.14 | 0.12 | 0.13 | 0.19 | 0.10 |
| **IA**  **Lar** | 0.06 | 0.07 | 0.09 | 0.08 | 0.07 | 0.07 | 0.08 | 0.08 | 0.06 | 0.07 | 0.06 | 0.09 | 0.10 | 0.07 | 0.10 | 0.12 | 0.10 | 0.10 | 0.11 | 0.11 | 0.11 | 0.12 | 0.10 | 0.08 | 0.09 | 0.09 | 0.10 | 0.08 | 0.09 | 0.11 | 0.08 | 0.05 | 0.06 | 0.15 | 0.13 | 0.11 | 0.11 | 0.15 | 0.08 |
| **IA**  **Lut** | 0.07 | 0.06 | 0.10 | 0.07 | 0.07 | 0.07 | 0.08 | 0.08 | 0.07 | 0.07 | 0.06 | 0.10 | 0.11 | 0.08 | 0.12 | 0.10 | 0.09 | 0.11 | 0.12 | 0.11 | 0.12 | 0.10 | 0.08 | 0.09 | 0.09 | 0.10 | 0.07 | 0.09 | 0.09 | 0.12 | 0.07 | 0.05 | 0.07 | 0.10 | 0.09 | 0.08 | 0.08 | 0.17 | 0.05 |
| **IL**  **Urb** | 0.10 | 0.10 | 0.13 | 0.11 | 0.10 | 0.10 | 0.11 | 0.11 | 0.10 | 0.10 | 0.11 | 0.14 | 0.14 | 0.11 | 0.15 | 0.14 | 0.13 | 0.15 | 0.15 | 0.15 | 0.15 | 0.14 | 0.12 | 0.13 | 0.13 | 0.13 | 0.11 | 0.11 | 0.13 | 0.16 | 0.10 | 0.08 | 0.10 | 0.16 | 0.13 | 0.11 | 0.13 | 0.19 | 0.10 |
| **NE**  **Con** | 0.06 | 0.07 | 0.09 | 0.08 | 0.07 | 0.07 | 0.08 | 0.08 | 0.06 | 0.07 | 0.06 | 0.09 | 0.10 | 0.07 | 0.12 | 0.10 | 0.10 | 0.11 | 0.11 | 0.11 | 0.12 | 0.10 | 0.08 | 0.09 | 0.09 | 0.10 | 0.07 | 0.08 | 0.09 | 0.11 | 0.08 | 0.05 | 0.06 | 0.15 | 0.12 | 0.09 | 0.11 | 0.15 | 0.08 |
